# Integrated multi-omic and symptom clustering reveals lower-gastrointestinal disorders of gut-brain interaction heterogeneity

**DOI:** 10.1080/19490976.2025.2604871

**Published:** 2025-12-23

**Authors:** Jarrah M. Dowrick, Nicole C. Roy, Caterina Carco, Shanalee C. James, Phoebe E. Heenan, Chris M. A. Frampton, Karl Fraser, Wayne Young, Janine Cooney, Tania Trower, Jacqueline I. Keenan, Warren C. McNabb, Jane A. Mullaney, Simone B. Bayer, Nicholas J. Talley, Richard B. Gearry, Timothy R. Angeli-Gordon

**Affiliations:** a Auckland Bioengineering Institute, University of Auckland, Auckland, New Zealand; b High-Value Nutrition National Science Challenge, Auckland, New Zealand; c Department of Human Nutrition, University of Otago, Christchurch, New Zealand; d Riddet Institute, Massey University, Palmerston North, New Zealand; e School of Food and Advanced Technology, Massey University, Palmerston North, New Zealand; f AgResearch, Palmerston North, New Zealand; g Department of Medicine, University of Otago, Christchurch, New Zealand; h Department of Psychological Medicine, University of Otago, Christchurch, New Zealand; i New Zealand Institute for Plant and Food Research Ltd., Hamilton, New Zealand; j Department of Surgery, University of Otago, Christchurch, New Zealand; k University of Newcastle, Callaghan, Australia; l Department of Surgery, University of Auckland, Auckland, New Zealand; m Te Manawahoukura Rangahau Centre, Te Wānanga o Aotearoa, Te Awamutu, Aotearoa, New Zealand

**Keywords:** Cluster analysis, disorders of gut-brain interaction, metagenomics, metabolomics, unsupervised machine learning, irritable bowel syndrome, functional constipation, functional diarrhea

## Abstract

Rome IV disorders of gut-brain interaction (DGBI) subtypes are known to be unstable and demonstrate high rates of non-treatment response, likely indicating patient heterogeneity. Cluster analysis, a type of unsupervised machine learning, can identify homogeneous sub-populations. Independent cluster analyses of symptom and biological data have highlighted its value in predicting patient outcomes. Integrated clustering of symptom and biological data may provide a unique multimodal perspective that better captures the complexity of DGBI. Here, integrated symptom and multi-omic cluster analysis was performed on a cohort of healthy controls and patients with lower-gastrointestinal tract DGBI. Cluster stability was assessed by considering how frequently pairs of participants appeared in the same cluster between different bootstrapped datasets. Functional enrichment analysis was performed on the biological signatures of stable DGBI-predominant clusters, implicating disrupted ammonia handling and metabolism as possible pathophysiologies present in a subset of patients with DGBI. Integrated clustering revealed subtypes that were not apparent using a singular modality, suggesting a symptom-only classification is prone to capturing heterogeneous sub-populations.

## Introduction

Disorders of gut-brain interaction (DGBI) are a collection of gastrointestinal (GI) disorders that lack an organic explanation but are, in part, driven by a breakdown in gut-brain communication.^1^ DGBI are currently diagnosed using the GI-symptom-based Rome IV diagnostic criteria.^2^ While the Rome IV criteria have enabled a collective scientific effort towards biomarker and treatment discovery, they have well-known limitations,^3^ including diagnostic instability^4^^,^^5^ and variable treatment responses.^6^ These limitations could indicate subtype heterogeneity, where patients with the same diagnosis have a differing underlying pathophysiology.^7^

The gut microbiome, a collective term for the microbial community that inhabits the GI tract, has been increasingly linked with pathophysiological phenomena, such as visceral hypersensitivity and disrupted GI barrier function, and could contribute to DGBI patient heterogeneity.^8^ Among its many other roles, the gut microbiome is essential for digestion, breaking down otherwise indigestible dietary fibers and complex carbohydrates.^9^ The resulting metabolites and other bioactive molecules are critical in mediating gut microbiome-host interaction.^9^ Metagenome and metabolome data provide insight into the functional capacity of the microbiome, the metabolic state of the host, and host-microbe interactions. Different phenotypes of the function and metabolic state of the gut microbiome may underpin subsets of lower-GI DGBI.

Cluster analysis is a form of unsupervised machine learning that can identify homogeneous patient groups.^10^ Symptom-based clustering has highlighted the value of psychological burden in distinguishing DGBI groups,^11^ predicting disease progression,^12^ and estimating healthcare costs.^13^ Biological cluster analyses have primarily focused on fecal microbiome samples, predicting microbiome-targeting treatment response.^14^ However, a limited number of studies have integrated metagenomic and metabolomic data,^15^ and the resulting clusters lacked stability, restricting deeper investigations into the underlying pathophysiology. Applications of modern cluster algorithms, capable of integrating rich biological and symptom datasets, may uncover latent patient subgroups and provide novel insights into lower-GI tract disease pathways that single-domain analyses might miss.

The present study was designed to uncover symptom-biology relationships that may inform larger-scale validation studies using a uniquely biologically rich dataset. A stepwise approach was used to investigate the heterogeneity of DGBI, which started with symptom data, followed by biological data, before integrating them into a unified cluster analysis. As clinical definitions should distinguish between health and disease, both controls and patients with DGBI were included in the cluster analyses. This approach enabled the data to indicate whether there was sufficient separation between clinical categories to naturally distinguish between groups. Functional enrichment analysis of the integrated clustering enabled an exploration of possible pathophysiological mechanisms driving symptom presentation.

## Materials and methods

### Study participants

Data used for cluster analysis was collected during the Christchurch IBS cOhort to investigate Mechanisms FOr gut Relief and improved Transit (COMFORT; 2016−2019).^16^ The recruitment process, clinical definitions, and exclusion and inclusion criteria have been reported previously,^16^ but are briefly summarized here.

COMFORT was a cross-sectional observation study comprised of participants between 18 and 70 years of age, recruited either from patients undergoing a colonoscopy at the endoscopy units in Christchurch, New Zealand, in response to advertisements or through direct contact with a member of the research team. Ethical approval was obtained from the Northern A Health and Disability Ethics Committee (16/NTA/21), and written informed consent was obtained from each participant before any study procedures were performed.

Recruited participants were excluded from the study if organic disease was discovered during colonoscopy or if they presented with “red flag” symptoms indicative of organic disease, including blood in the stool, nocturnal symptoms, unexplained weight loss, and anemia. Participants with a history of GI disease, abdominal surgery, pregnancy, familial polyposis syndromes, those who were unable to give informed consent, or with a positive fecal occult blood test or a calprotectin level >50 μg/g were also excluded.

Controls were defined as participants who did not meet the exclusion criteria^16^ and lacked significant GI symptoms. Controls recruited through colonoscopy did not have GI symptoms or a history of underlying significant health problems, including GI disease. Diagnostic labels of irritable bowel syndrome (IBS), functional constipation (FC), and functional diarrhea (FD) were assigned using the Rome IV questionnaire items.^17^ A total of 315 participants completed the study [129 controls, 57 diarrhea-predominant IBS (IBS-D), 30 constipation-predominant IBS (IBS-C), 41 mixed-type IBS (IBS-M), 42 FC, and 16 FD], and 14 additional participants partially completed the study but were not assigned a diagnostic label.

### Sample and data collection

Dietary data collection and questionnaires were completed over three days, with biological samples collected on the fourth day. Participants completed the Rome IV questionnaire,^17^ medical history, the Hospital Anxiety and Depression Scale (HADS),^18^ and the Structure Assessment of Gastrointestinal Symptoms (SAGIS).^19^ Concurrently, participants completed a Food And Symptom Times (FAST) diary, a validated three-day weighted food diary.^20^ On the third day, participants completed the Patient Reported Outcomes Measurement Information System (PROMIS) tools for GI symptoms^21^ and emotional distress.^22^ Exercise level was assessed using metabolic equivalent to tasks (MET), which were calculated as suggested by the Australian Bureau of Statistics.^23^

### Metagenomics

DNA extraction, shotgun metagenomics sequencing, and alignment of the COMFORT cohort samples have been described previously^24^ and are detailed in the Supplemental Methods. In brief, extracted DNA samples were paired-end shotgun sequenced, and libraries were prepared using the Illumina Nextera XT kit. The final library was denatured and sequenced on an Illumina NextSeq 550 using the NextSeq 500/550 High Output Kit v2.5 (300 Cycles). Raw sequencing data analysis was performed with Human Microbiome Project pipelines,^25^ and taxonomic classifications for COMFORT samples were determined using the SILVA 128 database.^26^ Sequences were then aligned against the NCBI non-redundant protein reference database using DIAMOND (v0.9.22) and subsequently assigned putative gene functions using MEGAN (v6 Ultimate Edition) and the Kyoto Encyclopedia of Genes and Genomes (KEGG) database (release 86.0). Taxa and gene abundance were scaled using total sum scaling, and those with an average abundance of less than 0.005% or less than 10% non-zero counts across participants were excluded from the cluster analysis. Environmental samples and unassigned taxa were also excluded.

The Firmicutes-Bacteroidetes phyla ratio was calculated by dividing the total sum score scaled phyla level taxonomic data of Firmicutes and Bacteroidetes. Average Shannon alpha diversity was calculated after rarefying to the minimum sampling depth in the dataset 100 times. Expected species richness was also estimated using the vegan R package (v2.6.8). These derived measures were used to compare clusters only.

### Untargeted metabolomics

Plasma and fecal samples were subjected to biphasic extraction, and global metabolite profiling was performed using polar, semi-polar, and non-polar untargeted liquid chromatography high-resolution mass spectrometry (LC-MS) methods as described previously.^27^ Raw MS data were processed with XCMS using R. Following peak extraction and alignment, data were normalized for batch and run-order effects using a loess algorithm with the Galaxy tool Workflow4Metabolomics. Metabolites with a relative standard deviation greater than 30% in the quality control samples were excluded from subsequent analysis. Detailed descriptions of the untargeted metabolomics workflow are provided in the Supplemental Methods.

### Targeted metabolomics

Targeted plasma amino acid^28^ and fecal bile acid^29^ metabolomics for the COMFORT cohort have been reported previously. Fecal short-chain fatty acids were measured using a modified MS-probe and stable LC-MS method.^30^ Plasma amino acids were measured using ultra-performance liquid chromatography analysis following a tungstate precipitation protocol.^28^ Detailed descriptions of the targeted metabolomics workflow are provided in the Supplemental Methods.

### Factor analysis

Bartlett's test of sphericity and Kaiser-Meyer-Olkin (KMO) test of factor adequacy were first performed to confirm the suitability of the COMFORT patient-reported outcome measures for factor analysis. A KMO index of at least 0.5 indicates sufficient shared variance to proceed with factor analysis,^31^ and a significance threshold of 0.05 was used for Bartlett's test of sphericity.

Factor analysis was then performed in Mplus (v8.10)^32^ using raw questionnaire item responses from HADS, SAGIS, and PROMIS T-scores for constipation, pain, diarrhea, difficulty swallowing, bloating, reflux, anxiety, and depression. Factor extraction was performed using the weighted least squares estimator, and the number of factors to retain was decided using Velicer's minimum average partial test via the EFA.dimensions R package (v0.1.8.4). Factors were rotated using geomin to enhance factor loading interpretability. Items with no loadings exceeding an absolute value of 0.4 were removed, and factor extraction was repeated. Cross-loading of items (*i.e.*, loading on multiple factors) was permitted. Factor names were assigned following manual inspection of the final rotated loadings. Factor scores were estimated using the maximum a posteriori method in Mplus.

### Cluster analysis

Robust clinical definitions should distinguish between healthy controls and patients with DGBI. To this end, both groups were included in all cluster analyses to assess whether identified clusters separated healthy controls from patients with DGBI.

#### Symptom clustering

Latent profile analysis (LPA) was performed on the derived factor scores using the tidyLPA R package (v1.1.0), with equal variances and covariances fixed to zero. The optimal number of profiles (hereon called clusters) was determined using bootstrap likelihood ratio testing with a significance threshold of 0.05. After fitting, LPA calculated participant-wise membership probabilities for each cluster. Each participant was assigned to the cluster with the greatest membership probability.

#### Biological clustering

Untargeted fecal and plasma metabolome, fecal metagenome (taxa and gene abundance), fecal bile, organic, and amino acid data were provided as input to the Neighborhood-based Multi-Omics clustering (NEMO; v0.1.0)^33^ pipeline in R (v4.4.3).^34^ Participants lacking at least one shared datatype were excluded, resulting in 307 participants being included in the biological clustering (123 controls, 39 FC, 15 FD, 30 IBS-C, 57 IBS-D, 36 IBS-M, and 7 with no diagnosis). The number of clusters was defined by NEMO.

#### Cluster stability

Cluster stability analysis was performed to assess how repeatable and robust the identified clusters were in response to dataset perturbations. First, a reference clustering was performed on the full dataset. Then, cluster analysis was repeated 1,000 times on bootstrapped datasets (75% of participants, sampled without replacement). For each iteration, two matrices were updated. The first, *M*, stored the number of times each pair of participants appeared in the bootstrapped datasets, and the second, *I*, stored the number of times each pair of participants was assigned to the same cluster. After all iterations were complete, a consensus matrix was computed by taking the element-wise division of *I* and *M*. A consensus value of 1 meant that pairs of participants were always clustered together if both were present in a bootstrapped dataset; a value of 0 meant that they were never clustered together.

To assess the stability of each cluster, we used the initial reference clustering to group the relevant consensus matrix elements. A cluster was considered stable if the mean or median consensus values exceeded 0.5, which was tested using a one-sample *t*-test or Wilcoxon signed-rank test, depending on normality assessed using the Shapiro-Wilk test. The 0.5 threshold aligns with validated criteria.^35^ Clusters not meeting this threshold were labeled unstable and not evaluated further.

#### Integrated symptom and biological clustering

The symptom and biological cluster analysis results were integrated using merged consensus clustering.^36^ An integrated consensus matrix was computed using the unweighted average of the symptom and biological consensus matrices calculated during stability analysis. Hierarchical cluster analysis (complete linkage) was performed, treating the integrated consensus matrix as a similarity measure. The optimal number of clusters was determined as the number associated with the lowest Davies-Bouldin Index (DBI) and highest silhouette score. The stability of clusters identified in the integrated cluster analysis was calculated using the integrated consensus matrix.

### Differential and functional analysis

Following integrated symptom and biological cluster analysis, a control-predominant cluster was designated as a reference group. The reference group was decided using symptom scores and the relative proportion of control participants. The metagenomic gene abundance and metabolomic data associated with each DGBI-predominant cluster were compared against this reference in differential expression analysis. Functional enrichment analysis was then performed on the resulting differentially expressed features.

#### Metagenomics

Metagenomic gene abundance was compared using the ALDEx2 R package (v1.36.0) with 128 Monte Carlo simulations. Benjamini-Hochberg corrected *P* values of Welch's t-test for each gene were used to determine differentially abundant genes (*P*_*adj*_ < 0.05). Gene enrichment analysis was then performed using the MicrobiomeProfiler R package (v1.10.0). The up- and down-regulated genes, as determined by ALDEx2 estimated effect size, were analysed separately.^37^ The background gene set was defined by all detected KEGG orthologs. *P* values were adjusted using the Benjamini-Hochberg procedure, and pathways were considered significant if *P*_*adj*_ was less than 0.05.

#### Metabolomics

Metabolomic data were compared using the R package Rvolcano (v1.0). Metabolites were defined as over- or underabundant if the Benjamini-Hochberg corrected robust *P* value was less than 0.05 and the kernel-weighted Log_2_ fold change was greater than 1 or less than −1, respectively. This analysis was performed separately for plasma metabolomics (untargeted and amino acids) and fecal metabolomics (untargeted, bile acids, and organic acids). Separate custom plasma and fecal reference metabolite sets were created using the identification conversion tool of MetaboAnalyst (v6.0) for all detected untargeted metabolites and assayed targeted metabolites.

Over-representation analysis was performed using MetaboAnalyst for overabundant and underabundant metabolites using the RaMP-DB pathway library. Topological pathway analysis was also performed in MetaboAnalyst with Fisher's exact test as the enrichment method, relative-betweenness centrality for the topology measure, and KEGG Homo sapiens (Dec 2023) for the pathway library. Following false discovery rate correction, a threshold of 0.1 was used to assign significance for over-representation and enrichment analysis. These analyses were performed separately for fecal (untargeted, bile acid, and organic acid) and plasma (untargeted and amino acid) metabolomics.

### Statistical analysis

Age was compared between controls and participants with a DGBI using a two-sample t-test, and the proportion of females between groups was compared with a chi-square test. The normality of cluster-wise measures was also assessed using the Shapiro-Wilk test, which was corrected for multiple comparisons using a Bonferroni adjustment. Measures were compared using one-way ANOVA or the Kruskal-Wallis test, depending on normality. Pairwise comparisons of significantly different, normally distributed variables were performed using Tukey's Honest Significant Difference. Dunn's test with a Benjamini-Hochberg post-hoc adjustment was used for the pairwise comparison of significantly different, non-normally distributed variables. Significance was assigned at the level of *P* < 0.05. Values are presented as mean ± standard deviation unless otherwise stated.

## Results

### Study cohort

**Figure 1.**
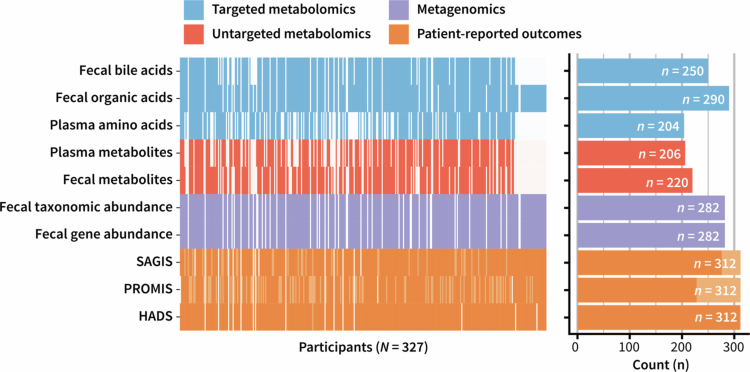
Data collected from the Christchurch IBS cohort to investigate mechanisms for gut relief and improved transit (COMFORT). Each column is associated with a single participant, and each row is a different measurement. Different base colors represent the different analysis pathways: blue—targeted metabolomics, red—untargeted metabolomics, purple—fecal metagenomics, and orange—patient-reported outcomes. Light elements indicate partial data, and empty elements indicate no data. The total samples for each measure are summarized in the bar chart to the right with the same color scheme.

The characteristics of the COMFORT cohort have been reported previously^16^ but are briefly repeated here for completeness. There was no significant difference in age between cases (53 ± 13 yrs) and controls (53 ± 12 yrs; *P* = 0.89), but there was a female (self-reported gender) predominance in the cohort (70%). The relative proportion of females was significantly different between the control participants (56%) and participants with DGBI (80%; *P* < 0.001). The measurements used in this present study are summarized in Figure 1.

### Factor analysis revealed distinct gut and psychological factors

The patient-reported outcome items had a KMO index of 0.883, and significance was obtained for Bartlett's test of sphericity (*P* < 0*.*001), indicating that these data were suitable for factor analysis. Velicer's minimum average partial test identified a seven-factor solution as optimal. After performing an initial factor extraction and rotation, two SAGIS questionnaire items (“excessive belching” and “loss of appetite”) were excluded as they did not have any absolute loadings exceeding 0.4. Repeating factor extraction and rotation with the revised patient-reported outcome items revealed a loading structure where every item was loaded onto at least one factor (Table S1). These factors were termed *Depression*, *Anxiety*, *Diarrhea*, *Nausea/vomiting*, *Pain/bloating*, *Upper GI*, and *Constipation*.

### Symptom clustering identified gut-centric and brain-centric clusters

**Figure 2.**
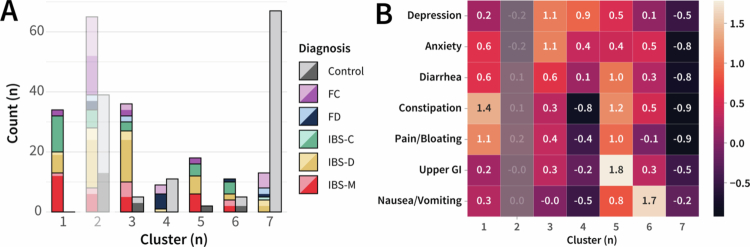
Symptom clustering identified gut-centric and brain-centric clusters. **(A)** Diagnostic breakdown underlying each latent profile (cluster). Colors denote the Rome IV diagnosis, with the left bar of each cluster breaking down the non-control participants and the right bar, the control participants. A darker shade for a given color represents participants with at least one gastrointestinal-related PROMIS score exceeding 55 (0.5 standard deviations above the population mean).^38^ **(B)** Heatmap indicating the scaled mean factor scores for each latent profile. A higher score was associated with a higher symptom burden for a given factor. Stability analysis indicated that Cluster Two was unstable, so its associated measures are included in a reduced opacity.

After estimating factor scores for the 315 participants who had at least partially completed symptom questionnaires, the bootstrap likelihood ratio test indicated that the optimal latent profile solution had seven clusters (Table S2). Cluster Two was deemed unstable (*P* = 0.650), so it was not considered further, but otherwise, the identified symptom-based clusters were stable (Fig. S1). No cluster was limited to a single Rome IV diagnosis (Figure 2A), though Cluster One only contained participants with DGBI, and Cluster Seven was most associated with healthy controls. These clusters differed in psychological and GI symptom burdens (Figure 2B).

Along with Cluster One, which consisted solely of DGBI participants, Clusters Three, Five, and Six also contained high percentages of DGBI participants (87.8%, 90%, and 68.8%, respectively). As expected, these clusters were associated with the greatest GI symptom burdens, each with unique combinations of symptoms. Cluster Three had a high psychological and moderate diarrhea symptom burden but much lower constipation and bloating compared to Cluster One, which had a lower psychological symptom burden. While generally greater in the high-percentage DGBI clusters, psychological symptom burden was not inextricably tied to GI-symptom burden. Cluster Four exhibited high psychological symptom scores in the absence of GI symptoms. Similarly, Cluster Three exhibited the greatest psychological burden but had lower GI symptom burden scores than Clusters One and Five.

Clusters Five and Six were associated with high upper-GI symptoms (*e.g.*, reflux) and nausea, respectively. Due to the potential for the derived factor scores to overstate the extent of upper-GI symptoms, the validated PROMIS T-scores for reflux and dysphagia were also considered. Upper GI and nausea/vomiting scores were also elevated in Clusters One and Three, albeit to a much lesser extent. Cluster Five had high average factor scores in each GI category.

### Biological clusters did not correspond with clinical definitions

**Figure 3.**
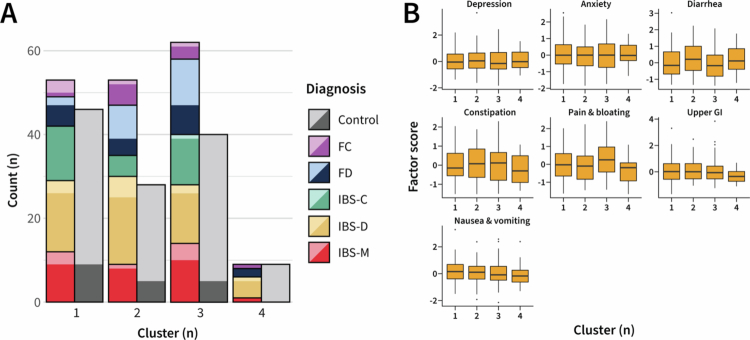
Biological clusters did not correspond with clinical definitions. **(A)** Diagnostic breakdown underlying each biological cluster identified by NEMO. Colors denote the Rome IV diagnosis, with the left bar of each cluster breaking down the non-control participants and the right bar, the control participants. A darker shade for a given color represents participants with at least one gastrointestinal-related PROMIS score exceeding 55 (0.5 standard deviations above the population mean).^38^ **(B)** Comparison of symptom factor scores between biological clusters. Depending on normality, as assessed using the Shapiro-Wilk test, differences in factor scores between clusters were evaluated using Bonferroni post-hoc corrected one-way ANOVA or Kruskal-Wallis test. Significance was assigned at the level of *P*_*adj*_ < 0.05.

The biological data types illustrated in Figure 1 were provided as input to NEMO, and four stable biologically informed clusters were obtained (Fig. S2). No apparent correlation was observed between biological cluster membership and the underlying Rome IV diagnostic categories (Figure 3A). This observation was supported by statistical analyses of factor scores, revealing no significant differences in any psychological or GI symptom factors across the identified clusters (Figure 3B).

### Integrated symptom and biological clusters

**Figure 4.**
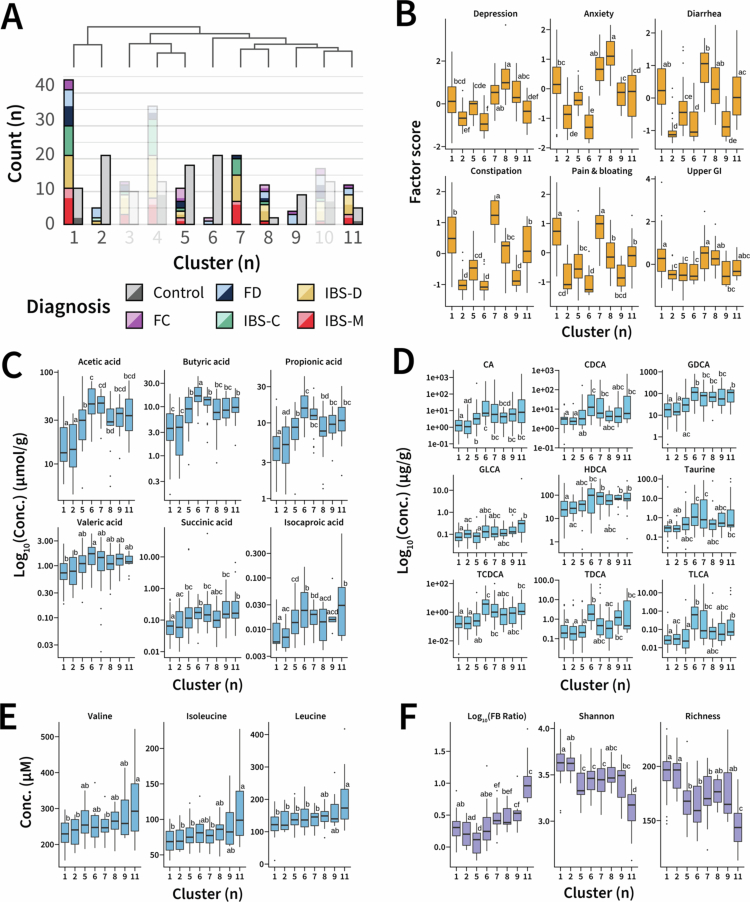
Characterization of integrated symptom-biological clusters. **(A)** Diagnostic breakdown underlying each biological cluster identified by NEMO. Colors denote the Rome IV diagnosis, with the left bar of each cluster breaking down the non-control participants and the right bar, the control participants. A darker shade for a given color represents participants with at least one gastrointestinal-related PROMIS score exceeding 55 (0.5 standard deviations above the population mean).^38^ Stability analysis indicated that Clusters Three, Four, and Ten were unstable, so their associated diagnosis counts are included in a reduced opacity. The dendrogram (upper) depicts the hierarchical relationships between the identified clusters based on average similarity profiles. **(B)** Comparison of symptom factor scores between biological clusters. **(C)** Comparison of fecal short-chain fatty acid concentrations between clusters. **(D)** Comparison of fecal bile acid concentrations between clusters. CA: cholic acid, CDCA: chenodeoxycholic acid, GDCA: glycoheoxycholic acid, GLCA: glycolithocholic acid, HDCA: hyodeoxycholic acid, TCDCA: taurochenodeoxycholic acid, TDCA: taurodeoxycholic acid, TLCA: taurolithocholic acid. **(E)** Comparison of plasma amino acid concentrations between clusters. **(F)** Comparison of microbial taxonomic dataset between clusters, including Firmicutes-Bacteroidetes phyla ratio (FB ratio), Shannon microbial alpha diversity, and expected microbial richness. For panels B-F, only features significantly different between clusters (one-way ANOVA or Kruskal-Wallis depending on normality) following Bonferroni post-hoc correction were included. Significance of pairwise comparisons using Tukey's Honest Significant Difference or Benjamini-Hochberg corrected Dunn test is denoted using compact letter display. Significance was assigned at the level of *P*_*adj*_ < 0.05.

A total of 300 participants were included in the integrated consensus matrix. The minimum DBI of 1.43 (Fig. S3A) and maximum silhouette score of 0.25 (Fig. S3B) each coincided with an 11-cluster solution. Eight of these clusters were stable (Fig. S3C). Clusters Three, Four, and Ten were not considered when comparing measurements due to their instability (*P* = 0.482*, P* = 0.962*, P* = 0.127, respectively). In merging the symptom and biological clustering results, clusters that varied in both their symptom burden and biological measures were obtained (Figure 4). No cluster was associated with a single bowel habit predominance among DGBI diagnoses (Figure 4A).

Control-predominant clusters (Two, Six, and Nine) had low symptom scores in almost every category (Figure 4B). In fact, Clusters Two and Six were symptomatically indistinguishable, with low symptom scores across all measures. Cluster Nine was similar but had significantly greater depression and anxiety than the other control-predominant clusters (*P =* 0.0406).

DGBI-predominant clusters (One, Seven, Eight, and Eleven) had varying symptom burden permutations. However, upper GI symptom scores were consistent (Figure 4B). Cluster Seven presented with the greatest constipation burden (*P* < 0.001) and high depression, anxiety, diarrhea, and pain/bloating symptom scores. The symptom profiles of Clusters One and Seven were similar, though Cluster One had less severe constipation (*P* < 0.001). Cluster Eight was notable for its high anxiety and depression scores, whereas Cluster Eleven presented the lowest symptom scores of the DGBI-predominant clusters.

From a biological perspective, Clusters Two and Six differed significantly in several measures despite equivalent symptom profiles. Cluster Six had greater concentrations of several fecal short-chain fatty acids (Figure 4C) and bile acids (Figure 4D) but did not differ in any of the plasma amino acid concentrations measured (Figure 4E). Cluster Two had greater expected richness (*P* < 0.001) and Shannon alpha diversity (*P* = 0.0296) (Figure 4F). Cluster Nine exhibited a greater Firmicutes-Bacteroidetes phyla ratio than the other control-predominant clusters (*P* = 0.0283) and differed from Cluster Two in acetic (*P* = 0.00373) and propionic acid (*P* = 0.0131) concentrations, as well as several bile acid concentrations.

DGBI-predominant Clusters Seven and Eight also lacked apparent biological features, but Clusters One and Eleven had several. Cluster Eleven had the lowest expected richness (*P* = 0.00118) and the highest Firmicutes-Bacteroidetes phyla ratio of the DGBI-predominant clusters (*P* = 0.0160). It also had raised concentrations of isoleucine, leucine, and valine relative to many other clusters (Figure 4E). Cluster One had increased microbial alpha diversity compared to Clusters Seven and Eleven (*P* = 0.00565) and generally low short-chain fatty acid and bile acid concentrations. There were no dietary or MET differences between the stable clusters.

### Functional analysis revealed distinct metabolic signatures

Cluster Six was selected as the reference group for differential expression analysis as it had the highest proportion of control participants and the joint lowest symptom scores (with Cluster Two). Of the DGBI-predominant clusters, only Clusters One and Eleven presented sufficiently different metagenomes or metabolomes from the reference cluster to perform functional analyses (Figs. S4−S13). To ease readability, Cluster One is hereinafter referred to as the Gut-Centric, High Pain Cluster, and Cluster Eleven is referred to as the Dysbiotic DGBI Cluster.

#### Gut-Centric, High Pain Cluster plasma metabolome exhibited altered ammonia handling

**Figure 5.**
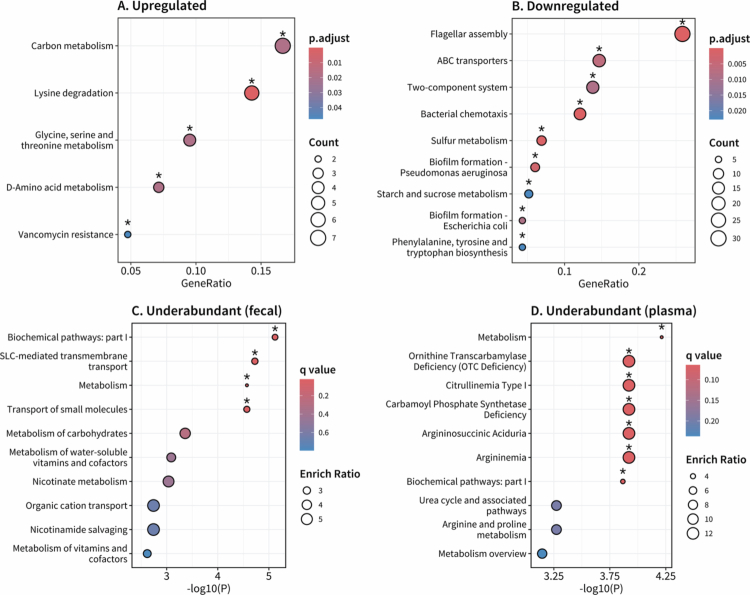
Gut-centric, high pain cluster (Cluster One) plasma metabolome exhibited altered ammonia handling. Enrichment analysis of **(A)** upregulated and **(B)** downregulated metagenome functional pathways and over-representation analysis of underabundant **(C)** fecal and **(D)** plasma metabolites. Differential expression was performed using Cluster Six as a reference group. Asterisks indicate significant pathways (*P*_*adj*_ < 0.05, metagenomics; *q* < 0.1, metabolomics).

The overabundant metagenomic functional pathways in the Gut-Centric, High Pain Cluster (Cluster One) were associated with carbon metabolism (*P* = 0.0192) and lysine degradation (*P* < 0.001; Figure 5A). Notable underabundant pathways were related to bacterial mobility (flagellar assembly and bacterial chemotaxis; *P* < 0.001), sulfur metabolism (*P* < 0.001), ATP-binding cassette transporters (*q* = 0.00610), and phenylalanine, tyrosine, and tryptophan biosynthesis (*q* = 0.0228; Figure 5B). Over-representation analysis of underabundant fecal and plasma metabolites highlighted disruptions to metabolism pathways (*q*_*fecal*_ = 0.0224, *q*_*plasma*_ = 0.0644). Fecal metabolites also implicated pathways associated with solute carrier-mediated transmembrane transport (*q* = 0.0224) and the transport of small molecules (*q* = 0.0224; Figure 5C). Plasma metabolites instead highlighted argininemia, citrullinemia, and ornithine transcarbamylase deficiency (*q* = 0.0644), though the urea cycle and associated pathways metabolite set was not significant (*q* = 0.197; Figure 5D). No significant pathways were obtained with the overabundant fecal metabolites, and insufficient overabundant plasma metabolites were identified to perform functional analysis. Topological functional analysis did not identify any significantly altered pathways.

#### Dysbiotic DGBI Cluster exhibited disrupted metabolism and transport

**Figure 6.**
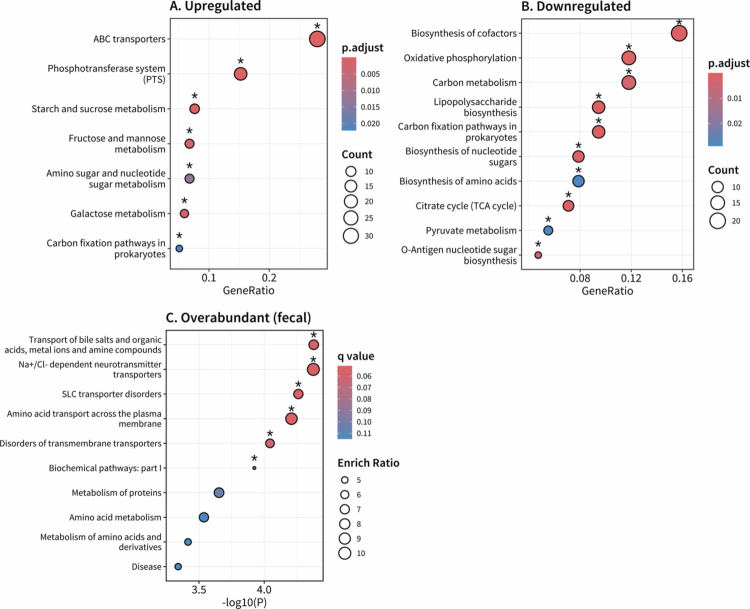
Dysbiotic DGBI cluster (Cluster Eleven) exhibited disrupted metabolism and transport. Enrichment analysis of **(A)** upregulated and **(B)** downregulated metagenome functional pathways. **(C)** Over-representation analysis of overabundant fecal metabolites. Differential expression was performed using Cluster Six as a reference group. Asterisks indicate significant pathways (*P*_*adj*_ < 0.05, metagenomics; *q* < 0.1, metabolomics).

The overabundant metagenomic functional pathways of the Dysbiotic DGBI Cluster (Cluster Eleven) were associated with ATP-binding cassette transporters (*P* < 0.001), phosphotransferase system (*P* < 0.001), starch and sugar metabolism, and carbon fixation (*P* = 0.0221; Figure 6A). Whereas underabundant functional pathways were related to the biosynthesis of cofactors, oxidative phosphorylation, carbon metabolism, and lipopolysaccharide biosynthesis (*P* < 0.001; Figure 6B). No differentially abundant plasma metabolites were detected, though there were several differentially abundant fecal metabolites (Fig. S9−S13). Over-representation analysis of overabundant fecal metabolites implicated several transport pathways, including disorders of transmembrane transporters (*q* = 0.0599), solute carrier transporter disorders (*q* = 0.0512), and transport of bile salts, organic acids, metal ions, and amine compounds (*q* = 0.0512; Figure 6C). Topological functional analysis of overabundant and underabundant fecal metabolites did not identify any significantly disrupted pathways.

## Discussion

This study reconsiders DGBI subtyping by applying unsupervised machine learning to a uniquely rich symptom and biological dataset. The resultant cluster analyses reiterate the heterogeneous nature of existing clinical definitions, reflecting the heterogeneity of treatment response of these conditions. Enrichment analysis of the underlying multi-omic data provides insights into possible DGBI phenotypes. The methods and results presented here present an alternative approach for categorizing patients into more homogeneous subpopulations, which, if expanded upon, could ultimately lead to improved diagnostic and treatment accuracy.

Symptom clustering indicated that upper and lower-GI symptoms can be present simultaneously, even among a cohort of participants with lower-GI DGBI and controls. In Rome IV, patients are restricted to a single diagnosis, and there are clear anatomical demarcations.^1^ These restrictions are contentious, however, with opposition mounting.^39^ When allowing multiple simultaneous DGBI diagnoses, or DGBI overlap, upper and lower DGBI often coincide in consistent permutations.^7^ Collectively, these data suggest that the underlying pathophysiology of lower-GI DGBI is not as regionally isolated as their name suggests.

None of the DGBI-predominant clusters identified here were associated with a single altered bowel habit (*i.e.*, diarrhea or constipation), contrasting with Rome IV. The present results contradict existing applications of finite mixture modeling to DGBI subtyping.^11^^,^^40^ However, key differences in the data used in the present study could explain this discrepancy. First, existing studies each included a small number of raw questionnaire item responses to indicate diarrhea and constipation, where here, factor analysis was used to estimate the underlying latent axis of constipation and diarrhea by consolidating three different validated GI-symptom questionnaires. Second, the cohort used in the present study included functional constipation, functional diarrhea, IBS, and control participants, instead of the IBS-only approach of past studies. Beyond the present cluster analysis results, assessments of bowel habit stability demonstrate that they are unreliable biomarkers,^41^ with patients fluctuating between classifications^42^ and bowel habits.^43^ Subjectivity of symptoms and potentially inconsistent standards of symptom assessments are challenges when using symptom data. In the present study, symptom scores were collected using validated patient-reported outcome questionnaires, including the ROME questionnaire. However, these challenges with symptom data further reiterate the need for actionable biomarkers in both research and clinical settings. Until physiological mechanisms are adequately understood, more pragmatic clinical pipelines that prioritize validated actionable biomarkers over patient-reported symptoms, such as those collated by Camilleri et al.,^44^ may prove more effective.

There is a spectrum of psychological symptom burdens present within patients with DGBI. Clusters were obtained with GI-predominant and psychological-predominant symptom profiles. Recent symptom-based clustering of IBS patients observed similar groups, referring to them as gut-mediated and brain-mediated clusters,^45^ confirming population-based studies.^46^ Speculatively, such clusters could aid in developing more targeted treatment strategies where the predominant symptom burden area is targeted first (*i.e.*, brain-centric vs gut-centric care).

Heterogeneity is not reserved solely for patients with a DGBI diagnosis, with multiple healthy subtypes also emerging. Multi-omic cluster analysis of asthma patients and healthy controls has also indicated multiple healthy subtypes.^47^ Given the reliance on healthy controls as a reference for traditional biomarker discovery, ignoring the possible subgroups within health will likely impede these approaches. Starting with cluster analysis should result in more homogeneous groups for comparison, albeit at the cost of sample size. Another challenge with clustering healthy subjects is that the researcher is forced to consider which cluster is the “healthiest” and, therefore, best suited to function as the reference group.^48^ In the present study, two clusters shared low symptom scores in all measures, so the relative proportion of control participants was used. An improved understanding of the underlying GI physiology will aid in a more specific, biologically informed definition of health for these applications.

Contrary to existing studies, a high Firmicutes-Bacteroidetes phyla ratio was not associated with a high symptom burden. Existing applications of cluster analysis to metagenome data have identified a high Firmicutes-Bacteroidetes phyla ratio as an indicator of a pathogenic-like microbiome signature among IBS patients.^14^^,^^15^^,^^49^ Patients with this signature have been more likely to respond to treatments that modify gut microbiome composition (*e.g.,* antibiotics and low FODMAP diet).^14^^,^^49^ A high Firmicutes-Bacteroidetes phyla ratio has also been implicated in inflammation and obesity,^50^^,^^51^ suggesting that it would be associated with a high symptom cluster. Here, however, the Dysbiotic DGBI Cluster presented with a high Firmicutes-Bacteroidetes phyla ratio but otherwise had relatively low pain symptom scores for a DGBI-predominant cluster. The other cluster presenting with a raised Firmicutes-Bacteroidetes phyla ratio was control-predominant. While this softens the explicit link between a high Firmicutes-Bacteroidetes phyla ratio and GI symptom presentation, the members of the control-predominant cluster may have additional compensatory mechanisms not present within the DGBI-predominant cluster. Further, the low-symptom burden does not preclude the possibility that members of the Dysbiotic DGBI Cluster could still benefit from gut microbiome-composition-centric treatment, though a follow-up study considering cluster-wise response rates is certainly necessary.

Functional analysis of DGBI-predominant clusters identified subgroups with distinct patterns of disruption. The Gut-Centric, High Pain Cluster exhibited a biological signature suggestive of altered microbiome-host amino acid and nitrogen metabolism. Gut microbes and the host compete for luminal amino acids,^52^ with the microbiota possessing an extensive capacity to catabolize and re-assimilate nitrogen from amino acids and ammonia.^53^^,^^54^ As a result, the increased microbial amino acid metabolic activity observed in this cluster may reduce the availability of amino acids to the host. Consistent with this possibility, plasma metabolomics revealed an underrepresentation of pathways annotated with urea-cycle-related disorders. These annotations likely reflect low levels of shared urea-cycle intermediates, such as ornithine, which has been reported to be reduced in IBS patients.^55^ Due to the role of urea-cycle intermediates in arginine and glutamate homeostasis, and glutamate is the precursor of GABA,^52^ a neurotransmitter thought to alleviate visceral hypersensitivity,^56^ it is possible that reduced host nitrogen flux contributes to the pain burden in this cluster. These observations suggest that a microbiota-induced low-nitrogen-flux metabolic state may contribute to lower-GI tract DGBI symptom burden, although mechanistic investigations are needed to confirm this hypothesis.

The Dysbiotic DGBI Cluster, on the other hand, was characterized by fecal metabolomic alterations in transport-related pathways and metagenomic disruptions in nutrient metabolism. Specifically, the enrichment of carbohydrate uptake mechanisms (ATP-binding cassette (ABC) transporters and the phosphotransferase system) and simple sugar metabolism, paired with the suppression of biosynthesis and energy-generation pathways, suggests the proliferation of fast-growing opportunistic taxa at the expense of metabolically independent, cooperative taxa. Although diet composition did not differ between clusters, these functional shifts parallel observations from high-sugar diet interventions, which have been associated with altered microbiome composition and low-grade inflammatory signaling.^57^^,^^58^ Strains with sugar ABC transporters have demonstrated enhanced growth and competitive advantage in complex consortia,^59^ which may have contributed to the observed dysbiosis. Fecal metabolomics indicated altered SLC transporter-mediated flux, consistent with disrupted intestinal barrier function in other GI conditions^60^^,^^61^ and metabolic disorders.^62^ Longitudinal studies are required to determine whether these functional signatures reflect exposure to a high-sugar diet, and to clarify the relationship between SLC transporter alterations and microbiome activity under these conditions.

The remaining DGBI-predominant clusters (Seven and Eight) did not present sufficient differences from the healthy-control reference cluster (Six), which may reflect the limited sample size or indicate that their pathophysiology involves mechanisms not captured by the current omics data, such as histological features or neural regulation.

Like the Rome Criteria, cluster analysis predicates itself on the assumption that different DGBI are discrete entities and not part of some continuum. Internal cluster validation indices provide some insight into the quality of a given clustering and, in some ways, indicate how “discrete” a collection of clusters is.^63^ For example, the silhouette score considers the relationship between intra-cluster and inter-cluster distance,^63^ with a maximum score of 1 indicating compact clusters that are well-separated. The maximum silhouette score for integrated biological and symptom clusters was approximately 0.25, which means a weak structure or poorly separated clusters.^64^ However, subsequent stability analysis was performed, and eight of the eleven merged clusters were found to be stable, suggesting sufficient separation that these clusters were repeatable and robust to data perturbation.

This study has notable limitations, including its cross-sectional nature and the modest sample size used for cluster analysis. Due to its cross-sectional design, we are unable to assess the longitudinal stability of cluster assignments or the temporal relationship between biological changes and symptom progression, thereby restricting our inferences of causality. The modest sample size may also limit the ability to detect rarer latent subgroups. However, several aspects of the data and methodology mitigate these concerns. Specifically, the dataset used was uniquely rich for each subject, which enabled detailed characterization that is often not captured in larger cohorts. Speaking to longitudinal stability, while GI symptom profiles are known to fluctuate,^42^ psychological symptoms are comparatively stable,^41^ and both fecal metagenome^65^^,^^66^ and fecal metabolome data^67^ exhibit lower within-person than between-person longitudinal variation. In addition, the use of consensus clustering enables the adjustment of relative weighting, or influence, of different measures and can be further adjusted in future validation work. Thus, although longitudinal investigation remains essential, the relative stability of key measures and adaptability of the methodology in the present study provides a strong foundation for future confirmational and clinical investigations.

To mitigate against the effects of a reduced sample size, a validated cluster stability method was used^35^ and only clusters robust to dataset perturbations were further evaluated. As the hypothesis for this analysis was that existing diagnostic labels are flawed, it was not possible to apply external validation methods.^68^ Instead, the combination of cluster stability testing and internal validation indices provided a rigorous methodological framework for evaluating cluster definitions in the absence of longitudinal data. While this framework supports the robustness of the present findings, future longitudinal and interventional studies will be critical to assess their clinical utility.

In conclusion, we performed integrated cluster analysis of multi-omic and symptom data collected from participants with lower-GI DGBI and control participants. This approach revealed latent clusters, a subset of which were interrogated for gut microbiome-host interaction mechanisms. These clusters emphasized the heterogeneous nature of DGBI, promoting a more pragmatic clinical approach informed by actionable biomarkers over the symptom-based Rome IV criteria.

## Supplementary Material

Supplementary material DGBI_clustering_SUPP_A_Figures_GutMicrobes

Supplementary material DGBI_clustering_SUPP_B_Extended_Methods

## Data Availability

Sequence reads for metagenomic sequencing are available for download from the National Center for Biotechnology Information (NCBI) Sequence Read Archive: https://www.ncbi.nlm.nih.gov/bioproject (accession number: PRJNA1088844). Metabolome data can be made available upon reasonable request to the lead contact. All original code has been deposited at Zenodo (https://www.doi.org/10.5281/zenodo.17148408) and is publicly available as of the date of publication.
