# Supplementary material for: Integrated multi-omic and symptom clustering reveals lower-gastrointestinal disorders of gut-brain interaction heterogeneity
Source: Gut Microbes. 2025 Dec 23;18(1):2604871. doi: 10.1080/19490976.2025.2604871 (PMC12758187; doi:10.1080/19490976.2025.2604871)
Supplement: Supplementary material — DGBI_clustering_SUPP_A_Figures_GutMicrobes [file KGMI_A_2604871_SM5692.pdf]

SUPPLEMENTAL MATERIALS A – Figures and Tables

Table S1: Rotated factor loadings from factor analysis using the Hospital Anxiety and Depression Scale (HADS), Patient-Reported Outcomes Measurement Information System (PROMIS), and Structured Assessment of Gastrointestinal Symptoms (SAGIS). Items with a loading greater than 0.4 were loaded onto a factor (bold).

Patient reported outcome items	Depression	Anxiety	Diarrhea/pain	Nausea/vomiting	Pain/bloating	Upper GI	Constipation
**D1 (HADS)**	**0.836**	-0.050	0.000	-0.050	-0.018	0.059	0.014
**D2 (HADS)**	**0.841**	-0.006	-0.089	0.068	0.134	0.013	-0.098
**D3 (HADS)**	**0.475**	0.103	0.144	-0.088	0.023	0.088	0.111
**D4 (HADS)**	**0.462**	0.093	0.047	-0.016	-0.096	0.182	-0.013
**D5 (HADS)**	**0.865**	0.007	-0.023	0.191	0.103	-0.085	0.030
**A6 (HADS)**	**0.572**	0.305	0.026	0.195	0.026	-0.105	0.058
**D6 (HADS)**	**0.912**	0.015	0.087	-0.141	-0.044	0.070	-0.059
**D7 (HADS)**	**0.497**	0.078	-0.148	0.233	-0.037	0.016	0.030
**Depression (PROMIS)**	**0.406**	0.383	0.036	-0.171	-0.048	0.185	0.099
**A1 (HADS)**	0.350	**0.563**	0.060	0.013	0.072	-0.073	-0.043
**A2 (HADS)**	0.012	**0.841**	0.014	-0.035	-0.059	0.114	-0.016
**A3 (HADS)**	-0.045	**0.848**	-0.026	0.017	0.040	0.143	-0.009
**A4 (HADS)**	0.186	**0.468**	-0.046	0.021	0.070	-0.179	-0.016
**A5 (HADS)**	0.273	**0.648**	0.054	0.055	-0.100	-0.068	0.026
**A7 (HADS)**	0.019	**0.877**	-0.033	0.042	0.018	-0.013	0.003
**Anxiety (PROMIS)**	0.097	**0.716**	-0.001	-0.116	0.048	0.173	0.017
**Diarrhea (PROMIS)**	0.031	0.017	**0.726**	-0.152	0.086	0.026	-0.049
**Pain or discomfort prior to bowel movement (SAGIS 8)**	-0.069	0.129	**0.476**	0.063	0.222	-0.029	0.312
**Loose stools (SAGIS 11)**	0.054	-0.081	**0.990**	-0.047	0.038	-0.043	-0.082
**Incontinence (SAGIS 12)**	0.065	-0.040	**0.551**	0.157	-0.256	0.079	0.257
**Urgency to empty the bowel (SAGIS 13)**	-0.028	-0.006	**0.845**	0.030	-0.054	0.012	0.086
**Diarrhoea (SAGIS 14)**	0.004	0.042	**0.956**	0.029	0.024	0.012	-0.151
**Sickness (discomfort combined with the impression for the need to vomit) (SAGIS 17)**	0.016	0.036	**0.432**	**0.672**	0.217	-0.005	0.029
**Nausea (urgent feeling of the need to vomit) (SAGIS 18)**	-0.016	0.101	0.367	**0.711**	0.164	0.059	-0.007
**Vomiting (vomiting of mucus and gastric contents, or strong unproductive retching) (SAGIS 19)**	0.018	-0.146	0.371	**0.642**	-0.037	0.210	0.010
**Fullness (feeling of congestion of food without relation to prior food intake) (SAGIS 3)**	0.136	-0.087	-0.053	0.012	**0.744**	0.216	0.010
**Early satiety (stomach is overfilled soon after starting to eat, disproportional to the quantity of food taken, so that food cannot be finished) (SAGIS 4)**	-0.006	0.028	0.085	0.098	**0.535**	0.318	-0.006
**Postprandial pain or discomfort (upper abdominal symptoms start or get worse after meals) (SAGIS 5)**	-0.006	-0.027	0.032	0.031	**0.735**	0.356	-0.024
**Epigastric pain/upper abdominal pain (pain between the belly button and chest/pain noticeable in the upper abdomen) (SAGIS 6)**	-0.044	0.020	-0.022	0.127	**0.684**	0.393	-0.040

**Abdominal cramps (spasmodic or colic like stomach pain without specified localisation) (SAGIS 16)**	-0.149	0.130	0.333	0.084	**0.437**	0.048	0.119
**Bloating (feeling of distension and excessive gas in the abdomen) (SAGIS 20)**	0.063	0.018	0.321	-0.136	**0.737**	-0.087	0.067
**Excessive gas and passing of wind (SAGIS 21)**	0.019	0.049	0.313	-0.224	**0.556**	0.003	0.101
**Pain (PROMIS)**	0.010	0.059	0.186	0.014	**0.598**	0.009	0.094
**Bloating (PROMIS)**	-0.005	-0.018	0.211	-0.142	**0.644**	0.014	0.102
**Retrosternal discomfort (unpleasant feeling behind the middle of the chest, painful or drawing) (SAGIS 7)**	0.060	-0.048	-0.040	0.153	**0.446**	**0.530**	-0.034
**Belching with acid taste/heartburn/burning sensation in the esophagus (SAGIS 1)**	-0.025	-0.018	0.055	0.002	0.268	**0.458**	0.147
**Dysphagia (difficulty swallowing) (SAGIS 2)**	0.019	0.021	0.001	0.090	0.026	**0.698**	0.051
**Reflux (PROMIS)**	0.003	0.084	0.055	-0.088	0.145	**0.645**	0.036
**Dysphagia (PROMIS)**	0.052	0.066	0.014	0.006	0.007	**0.585**	0.078
**Constipation (SAGIS 10)**	0.035	-0.019	-0.210	0.009	0.065	0.007	**0.948**
**Difficulty with emptying the bowel (SAGIS 9)**	0.009	0.008	0.134	-0.090	0.013	-0.014	**0.869**
**Constipation (PROMIS)**	-0.012	0.049	-0.004	0.036	0.039	0.054	**0.751**

Table S2: Bootstrap likelihood ratio test results for determining the optimal number of profiles for latent profile analysis. LRTS – likelihood ratio test statistic.

Comparison	LRTS	*P*-value
1 vs 2	414.23	0.001
2 vs 3	94.44	0.001
3 vs 4	35.46	0.001
4 vs 5	54.15	0.001
5 vs 6	26.46	0.018
6 vs 7	36.67	0.002
7 vs 8	21.5	0.055

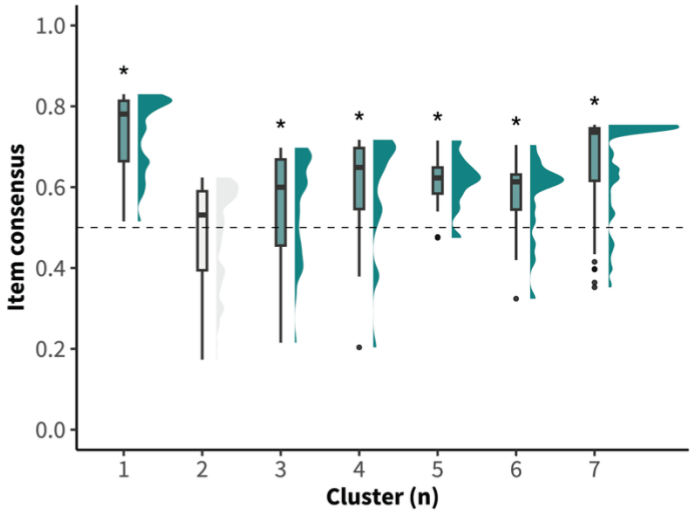

Figure S1: Symptom clustering stability. A higher consensus score indicates that a given participant was consistently grouped with the same participants. Clusters significantly greater than the stability threshold of 0.5 ($P<0.05$) are indicated with an asterisk and teal coloration.

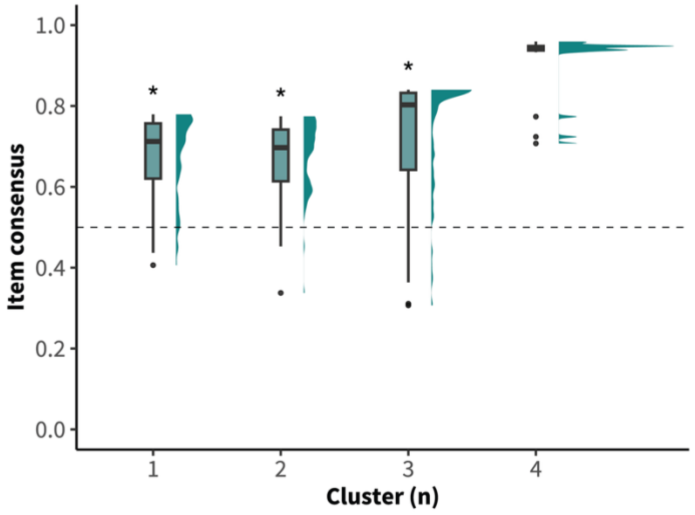

Figure S2: Biological clustering stability. As described in Fig. S1.

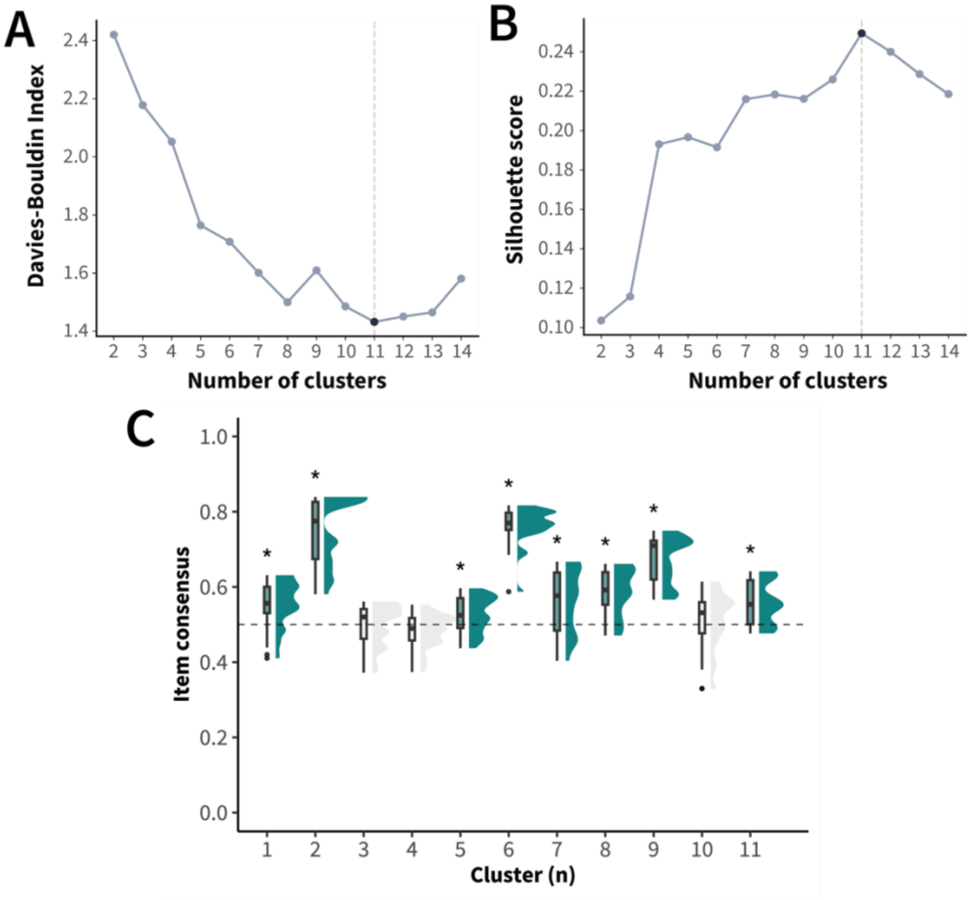

Figure S3: Merged biological-symptom clustering. (A) Davies-Bouldin Index (DBI) and **(B)** silhouette score for clustering solutions with between two and fourteen clusters. A lesser DBI and greater silhouette score reflect a better clustering solution. Optimal clustering solution is indicated by a vertical dashed line. **(C)** Item-wise consensus for each cluster. As described in Fig. S1.

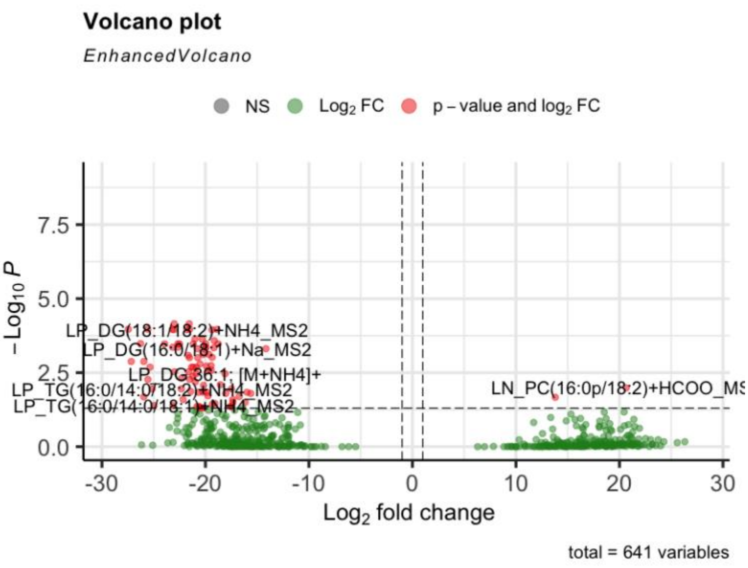

Figure S4: Robust volcano analysis comparing untargeted plasma metabolomic data between the Gut-Centric, High Pain Cluster (One) and the Healthy Reference Cluster (Six). Metabolites that were significantly different, as determined by a Benjamini-Hochberg corrected *P* value <0.05 and exhibited a kernel-weighted $\log_2$-fold change greater than 1 or less than -1, are located in the upper left and upper right areas of the plot and colored red. Of the 641 metabolites included in the differential analysis, 83 were downregulated and 2 were upregulated relative to the Healthy Reference Cluster. Significant metabolites were included in a downstream functional enrichment analysis, the results of which are included in Figure 5.

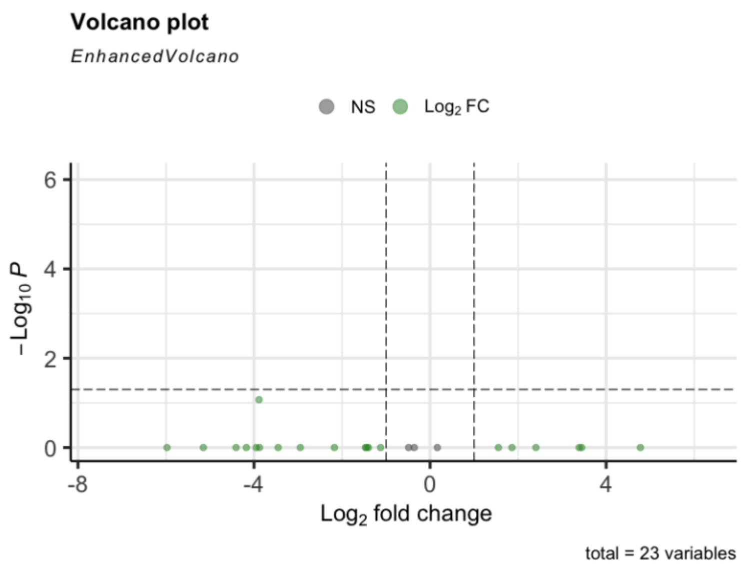

Figure S5: Robust volcano analysis comparing plasma amino acids between the Gut-Centric, High Pain Cluster (One) and the Healthy Reference Cluster (Six). While several plasma amino acids exhibited a kernel-weighted $\log_2$-fold change greater than 1 or less than -1, none were significantly different (Benjamini-Hochberg corrected *P* value<0.05), so none were included in the downstream functional analyses.

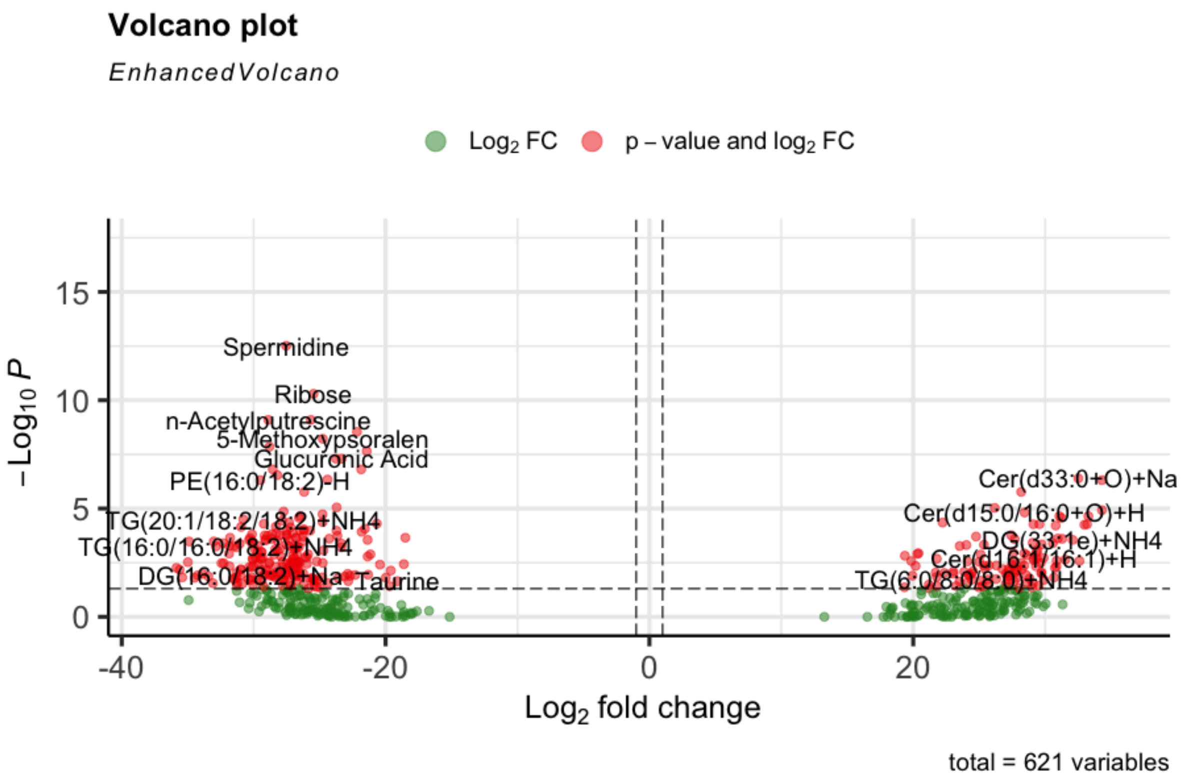

Figure S6: Robust volcano analysis comparing untargeted fecal metabolomic data between the Gut-Centric, High Pain Cluster (One) and the Healthy Reference Cluster (Six). Metabolites that were significantly different, as determined by a Benjamini-Hochberg corrected *P* value <0.05 and exhibited a kernel-weighted $\log_2$-fold change greater than 1 or less than -1, are located in the upper left and upper right areas of the plot and colored red. Of the 621 metabolites included in the differential analysis, 220 were downregulated and 111 were upregulated. Significant metabolites were included in a downstream functional enrichment analysis, the results of which are included in Figure 5.

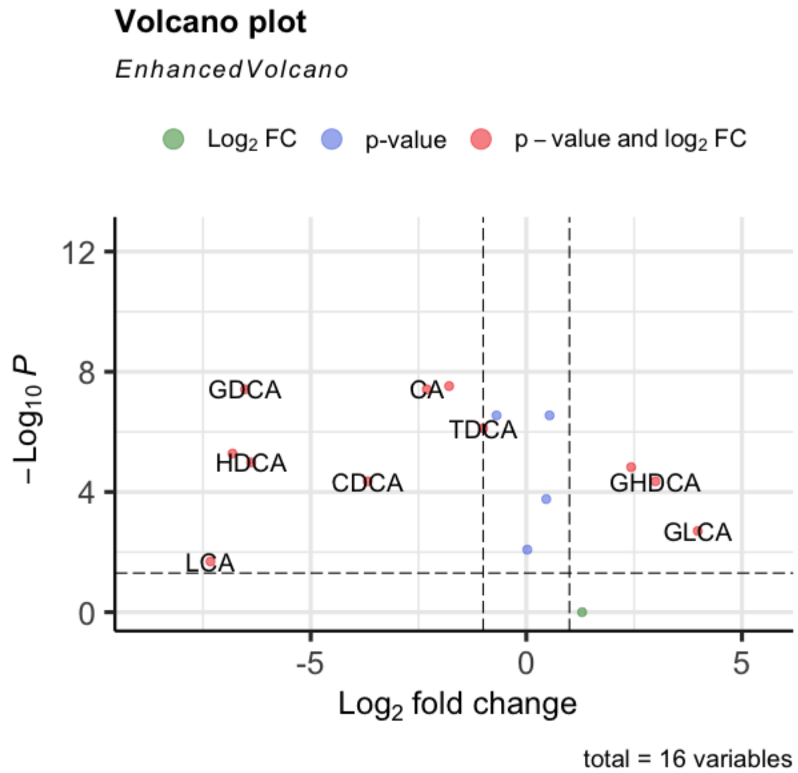

Figure S7: Robust volcano analysis comparing fecal bile acids between the Gut-Centric, High Pain Cluster (One) and the Healthy Reference Cluster (Six). Metabolites that were significantly different, as determined by a Benjamini-Hochberg corrected *P* value <0.05 and exhibited a kernel-weighted $\log_2$-fold change greater than 1 or less than -1, are located in the upper left and upper right areas of the plot and colored red. Of the 16 bile acids included in the differential analysis, 8 were downregulated and 3 were upregulated. Significant metabolites were included in a downstream functional enrichment analysis, the results of which are included in Figure 5. GDCA: glycodeoxycholic acid, HDCA: hyodeoxycholic acid, CDCA: chenodeoxycholic acid, LCA: lithocholic acid, CA: cholic acid, TDCA: taurochenodeoxycholic acid, GHDCA: glycohyodeoxycholic acid, GLCA: glycolithocholic acid.

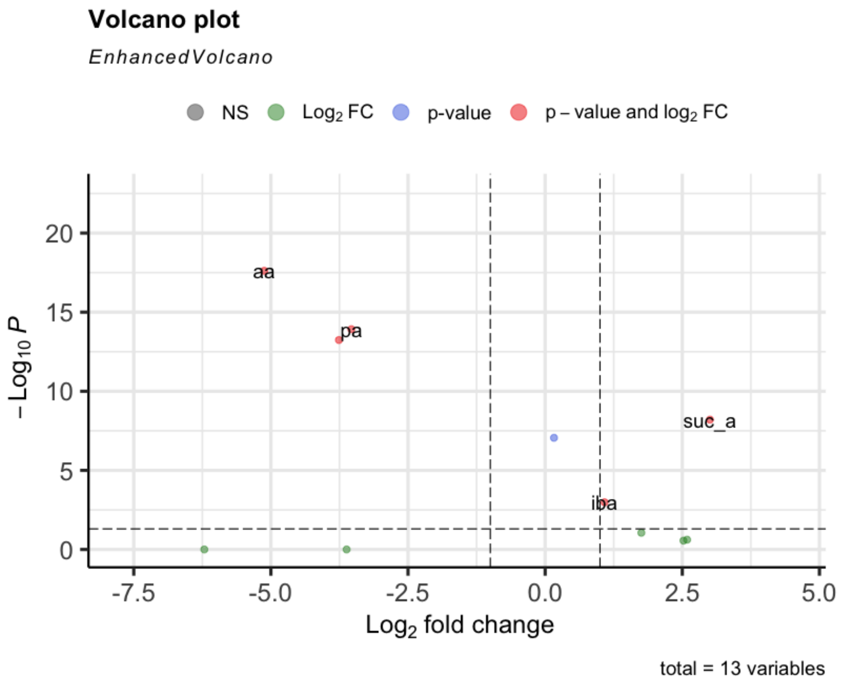

Figure S8: Robust volcano analysis comparing fecal short-chain fatty acids between the Gut-Centric, High Pain Cluster (One) and the Healthy Reference Cluster (Six). Metabolites that were significantly different, as determined by a Benjamini-Hochberg corrected *P* value <0.05 and exhibited a kernel-weighted log_2-fold change greater than 1 or less than -1, are located in the upper left and upper right areas of the plot and colored red. Of the 13 short-chain fatty acids included in the differential analysis, 3 were downregulated and 2 were upregulated. Significantly different abundant metabolites were included in a downstream functional enrichment analysis, the results of which are included in Figure 5. aa: acetic acid, pa: propionic acid, iba: isobutyric acid (2-methylpropanoic acid): suc_a, succinic acid.

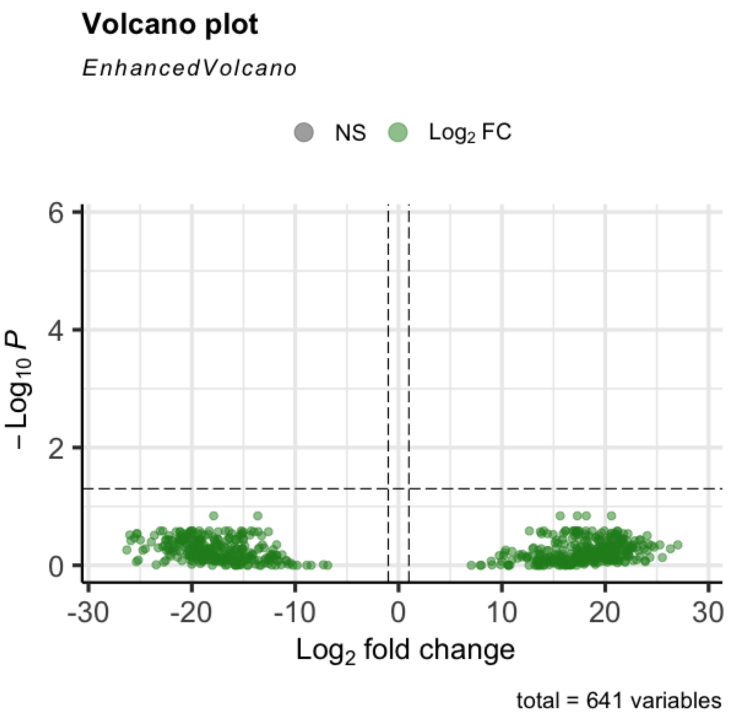

Figure S9: Robust volcano analysis comparing untargeted plasma metabolomics between the Dysbiotic DGBI Cluster (Eleven) and the Healthy Reference Cluster (Six). While all plasma metabolites exhibited a kernel-weighted $\log_2$-fold change greater than 1 or less than -1, none were significantly different (Benjamini-Hochberg corrected *P* value<0.05), so none were included in the downstream functional analyses.

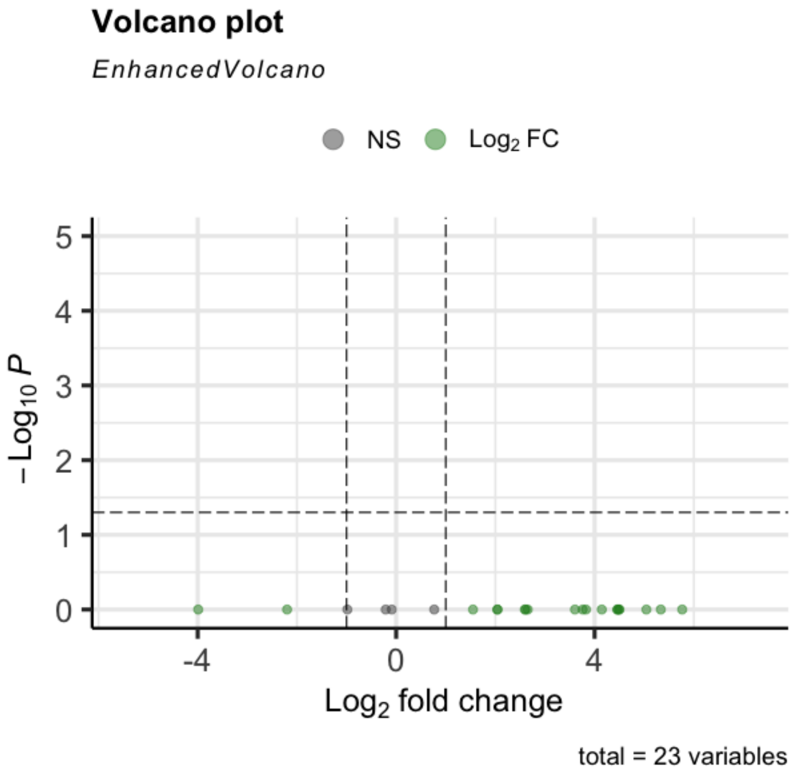

Figure S10: Robust volcano analysis comparing plasma amino acids between the Dysbiotic DGBI Cluster (Eleven) and the Healthy Reference Cluster (Six). While several plasma amino acids exhibited a kernel-weighted $\log_2$-fold change greater than 1 or less than -1, none were significantly different (Benjamini-Hochberg corrected *P* value<0.05), so none were included in the downstream functional analyses.

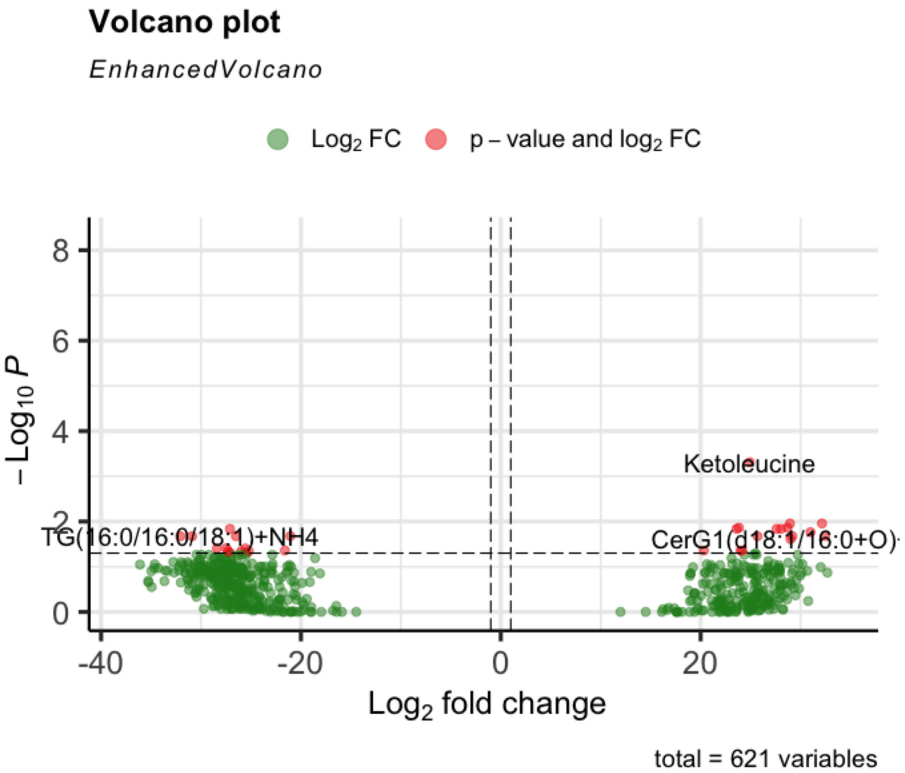

Figure S11: Robust volcano analysis comparing untargeted fecal metabolomic data between the Dysbiotic DGBI Cluster (Eleven) and the Healthy Reference Cluster (Six). Metabolites that were significantly different, as determined by a Benjamini-Hochberg corrected *P* value <0.05 and exhibited a kernel-weighted $\log_2$-fold change greater than 1 or less than -1, are located in the upper left and upper right areas of the plot and colored red. Of the 621 metabolites included in the differential analysis, 13 were downregulated and 16 were upregulated. Significant metabolites were included in a downstream functional enrichment analysis, the results of which are included in Figure 6.

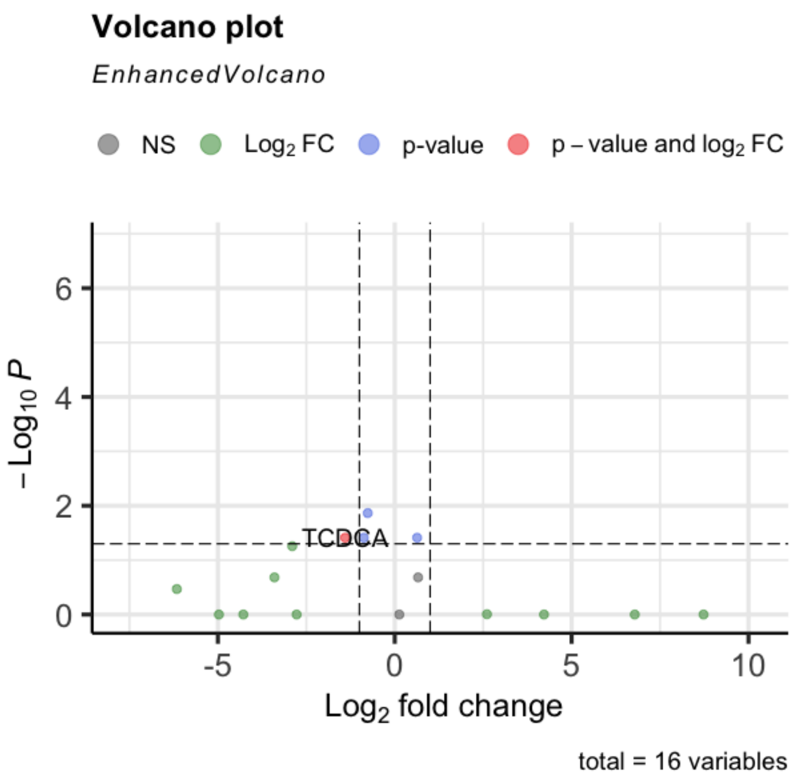

Figure S12: Robust volcano analysis comparing fecal bile acids between the Dysbiotic DGBI Cluster (Eleven) and the Healthy Reference Cluster (Six). Metabolites that were significantly different, as determined by a Benjamini-Hochberg corrected *P* value <0.05 and exhibited a kernel-weighted log_2-fold change greater than 1 or less than -1, are located in the upper left and upper right areas of the plot and colored red. Of the 16 bile acids included in the differential analysis, only taurochenodeoxycholic acid (TCDCA) was downregulated, and none were upregulated. Significant metabolites were included in a downstream functional enrichment analysis, the results of which are included in Figure 6.

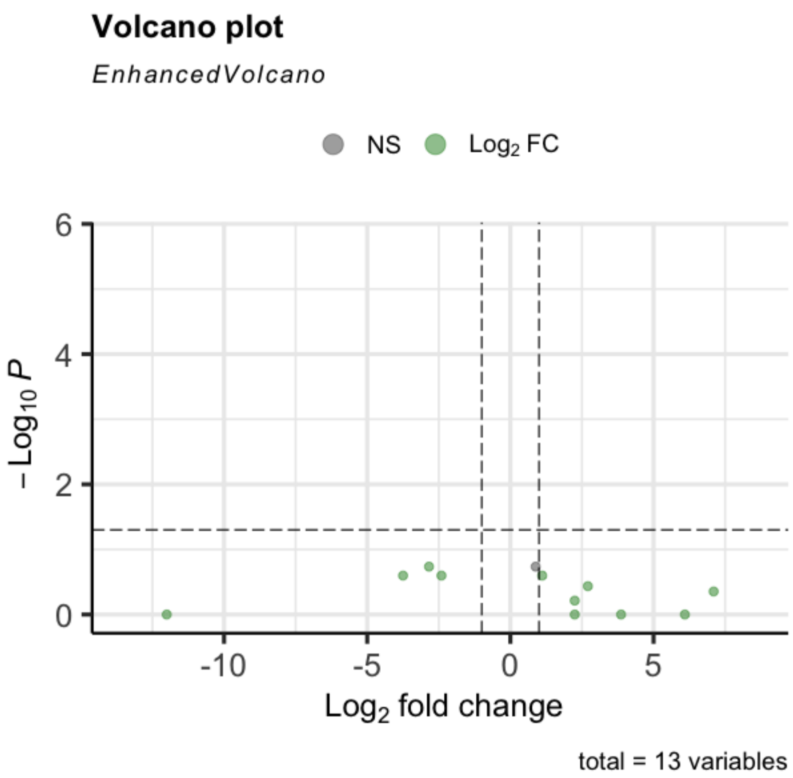

Figure S13: Robust volcano analysis comparing fecal short-chain fatty acids between the Dysbiotic DGBI Cluster (Eleven) and the Healthy Reference Cluster (Six). While several fecal short-chain fatty acids exhibited an absolute kernel-weighted $\log_2$-fold change greater than 1, none were significantly different (Benjamini-Hochberg corrected *P* value<0.05), so none were included in the downstream functional analyses.
