# Supplementary material for: Integrated multi-omic and symptom clustering reveals lower-gastrointestinal disorders of gut-brain interaction heterogeneity
Source: Gut Microbes. 2025 Dec 23;18(1):2604871. doi: 10.1080/19490976.2025.2604871 (PMC12758187; doi:10.1080/19490976.2025.2604871)
Supplement: Supplementary material — DGBI_clustering_SUPP_B_Extended_Methods [file KGMI_A_2604871_SM5691.docx]

**SUPPLEMENTAL MATERIALS B – Extended Materials and Methods**

# Sample collection and storage

Following the collection of written informed consent, an electronic case report form was generated for each participant. In creating this report form, participants were deidentified and assigned a unique fivedigit numerical identifier. ID-labelled questionnaires and biological sample collection equipment were posted to participants. All data and biological samples were collected from participants undergoing colonoscopy before the commencement of bowel preparation. Collected questionnaire responses and biological samples were stored physically or electronically in accordance with ethical requirements.

Participants were asked to collect a fecal sample at home, store it at -20 ^o^C, and deliver it to the research team within 24 hours of collection. Upon arrival with their fecal sample, a blood sample was collected from participants. Two aliquots each containing 1 g of fecal material were snap-frozen in liquid nitrogen and stored at -80 ^o^C until extraction. Lithium Heparin-treated blood (18 mL), Ethylenediaminetetraacetic Acid (12 mL) treated blood, and untreated blood (10 mL) samples were centrifuged at 2000× g, and plasma was distributed into 0.5 mL aliquots and stored at -80 °C.

# Metagenomics

## DNA extraction

DNA was extracted using the Macherey-Nagel NucleoSpin Soil kit (Macherey-Nagel, Auckland, New Zealand). First, approximately 100-200 mg of the sample was transferred to a tube containing ceramic beads, and the weight was recorded. Next, 700 μL of buffer SL2 was added, and the sample was vortexed briefly. Following a 20-minute incubation at room temperature, 150 μL of Enhancer SX was added to each tube to lyse the material. The samples were then homogenised for 4 min at 3000 rpm using the Mini Bead-beater 96 (Biospec, Gisborne, New Zealand).

After the bead-beating, particulate matter was precipitated by centrifuging the samples at 11,000× g for 2 min at room temperature. The clear supernatants were then transferred to a new collection tube.

150 μL of lysis buffer SL3 was added, followed by vortexing for 5 s, and incubating for 5 min at 4 ˚C. Next, the tube was centrifuged at 11,000× g for 3 min at room temperature, and clear supernatant was loaded into a new collection tube with a red NucleoSpin Inhibitor Removal Column filter. After centrifuging the tubes at 11,000× g for 1 min at room temperature, the column was discarded, and 250 μL of binding buffer SB was added to the flow-through. Next, the sample was mixed by inversion and loaded onto a green NucleoSpin Soil Column with a new collection tube. The sample was then centrifuged at 11,000× g for 1 min at room temperature, and the flow-through containing host DNA was discarded. A further 500 μL of SB buffer was added, and the sample centrifuged again, discarding flow through. 550 μL of the wash buffer SW1 followed, then 700 μL of the wash buffer SW2 was used for the second wash. Another 700 μL of the wash buffer SW2 was added, and the tubes were left to sit for 20 min at room temperature. Tubes were centrifuged again at 11,000× g for 30 s at room temperature. One more centrifugation step without wash buffer was performed to dry the column, and then 30 μL of elution buffer was added to the column, which was left to sit at room temperature for 2 min. The microbial DNA was then eluted by centrifuging at 11,000× g for 1 min at room temperature. The eluted DNA was transferred back to the same column and centrifuged again to optimize recovery.

The microbial DNA was quantitated using the NanoDrop analyser (Thermofisher Scientific, Waltham, USA) and checked for fragmentation induced by bead beating by visualising 2 μL of DNA on a 1-2% agarose gel. DNA was stored at -80 ^o^C. DNA samples met the sequencing criteria from Teagasc, as determined by the amount of DNA (0.2 ng/μL in 5 μL) and the DNA quality (260/280 ratio > 1.5).

## Shotgun metagenomic sequencing

The DNA shotgun libraries were prepared using the Illumina Nextera XT kit, as described in the manufacturer’s instructions, with the exception that tagmentation time was increased from 5 to 7 min. Following index PCR and cleanup, the sizes of the fragments in all samples were assessed by running on an Agilent bioanalyser using an Agilent High Sensitivity Kit and quantified using a Qubit High Sensitivity Kit. The libraries were then pooled equimolarly, and the final concentration was determined using the Kapa Library Quantification Kit for Illumina. The final library was then denatured and sequenced on an Illumina NextSeq 550 using the NextSeq 500/550 High Output Kit v2.5 (300 Cycles) as outlined in the manufacturer’s instructions.

## Data acquisition

Paired sequences were merged using PEAR version 0.9.6.^1^ Host sequences were detected and removed using the BBMAP package version 38.22-0 with the human genome (Human GRCh38) as a reference. Metaxa2 version 2.1.3a^2^ identified small-subunit ribosomal DNA, and taxonomic classifications were determined using the Silva 128 database.^3^ Next, sequences were aligned against the NCBI nonredundant protein reference database using the "blastx" function of DIAMOND version 0.9.22.^4^ Finally, the DIAMOND alignment files were assigned with putative gene functions using MEGAN version 6 Ultimate Edition^5^ against the Kyoto Encyclopedia of Genes and Genomes (KEGG) database.

# Untargeted metabolomics

Methods and data are reported in Fraser et al. (2024).^6^

***Standards and reagents:***

Standards and Reagents Internal standards d4-alanine, d2-tyrosine, d5-tryptophan, and d10-leucine were purchased from Cambridge Isotope Laboratories, Inc. (Tewksbury, MA, USA) as standards for monitoring polar and semi-polar LC-MS performance. The lipidomics internal standard, 1-palmitoyld31-2-oleoyl-sn-glycero-3-phosphate (PE 16:0 D31/18:1 sodium salt, for monitoring the lipidomics LC-MS performance) was purchased from Avanti® Polar Lipids, Inc. (Birmingham, AL, USA), while ammonium formate and formic acid were purchased from Sigma Aldrich (Auckland, New Zealand). Acetonitrile, methanol, methyl tert-butyl ether, and chloroform of optima LC-MS grade were purchased from Thermo Fisher Scientific (Auckland, New Zealand).

## Plasma sample extraction

Plasma samples were extracted using biphasic extraction, slightly adapted from a previously reported method.^7^ Briefly, 100 μL plasma in a microcentrifuge tube was mixed with 800 μL pre-chilled (−20 °C) CHCl3:MeOH (50:50, v/v) containing 10 μg/mL of the internal standards d4-alanine, d2-tyrosine, d5tryptophan, and d10-leucine. The sample was then agitated for 30 s and stored at −20 °C for 60 min to allow protein precipitation, followed by the addition of 400 μL H_2_O, vortex mixed for 30 s, and then centrifuged at 14,000× g, 4 °C, for 10 min. Blank samples were prepared following the same protocol, replacing plasma with H_2_O. Two hundred microlitres of the upper aqueous layer was transferred to a tube for polar analysis, a further 200 μL was transferred to another tube for semi-polar analysis, and 200 μL of the lower organic layer was transferred to a tube for lipidomic analysis.

All tubes were evaporated to dryness under a nitrogen stream and stored at −80 °C. To account for intra- and inter-batch variation, pooled quality control (QC) samples were prepared by combining an aliquot of the upper or lower phase from every sample extracted on the same day in a clean glass tube and stored at −80 °C for each of the three metabolomic streams. At the end of all sample extractions, the pooled samples from each day were combined, dispensed into separate 200 μL aliquots, and evaporated to dryness under a nitrogen stream. The samples were then stored at −80 °C. On the day of instrumental analysis of the plasma extracts, dried polar, semi-polar, and lipid extracts were reconstituted in 200 μL acetonitrile:H_2_O (50:50, v/v), 200 μL acetonitrile:H_2_O (10:90, v/v), and 200 μL modified Folch solution (CHCl3:MeOH:H_2_O, 66:33:1, v/v/v) containing pre-dissolved 0.01% PE(16:0 D31/18:1) internal standard [0.01% (%w/v)] for polar, semi-polar, and lipid metabolite analyses, respectively, and transferred to glass HPLC vials containing 250 μL glass inserts.

## Fecal sample extraction

Fecal samples were extracted using a biphasic extraction method slightly adapted from a previously reported method.^8^ Briefly, samples were freeze-dried under vacuum, ground, and 50 mg was weighed and transferred to 2.0 mL microcentrifuge tubes with a ceramic bead for further homogenisation for 1 min using a QIAGEN TissueLyser II (Thermo Fisher Scientific, Auckland, New Zealand). Next, 400 µL of 75% MeOH/MilliQ H_2_O was added, and the tubes were vortexed for 30 s. The samples were sonicated for 2 min and then transferred onto ice for 10 min. Next, 1 mL of MTBE was added, and the samples were agitated on a shaker for 1 h at 4 °C and 450 rpm. MilliQ water (250 µL) was added, and the samples were vortexed for 30 s and left to rest for 10 min. Tubes were centrifuged (Eppendorf Centrifuge 5427 R, Eppendorf, Hamburg, Germany) at 14,000× g for 25 min at 4 °C, 850 µL of the upper lipid phase was transferred to a new tube to be used for lipid analysis, and 300 µL of MilliQ water was added to the remaining extract and then vortex mixed for 30 s and centrifuged for a further 20 min (14,000× g, 4 °C). The remaining polar phase was transferred to a new tube and centrifuged for a further 20 min to remove any fine particles. Then, 300 µL aliquots of the polar phase were transferred into two different tubes for polar and semi-polar metabolite analyses. Finally, the extracts from all three microcentrifuge tubes were evaporated to dryness under nitrogen. On the day of instrumental analysis of the fecal extracts, samples for lipidomic analysis were reconstituted in 500 µL of a 2:1 CHCl_3_:MeOH containing PE(16:0 D31/18:1) internal standard at 10 µg/mL concentration and vortexed until all material was redissolved. The samples were centrifuged at 12,000× g for 12 min at 4 °C, and 100 µL of the solution was transferred to a glass HPLC vial containing a 250 µL insert. Polar fecal extracts for metabolomic analysis using HILIC were reconstituted in 200 µL of a 50:50 acetonitrile/H_2_O solution and then vortexed until all material dissolved. The extracts were centrifuged at 12,000× g for 12 min at 4 °C, and 100 µL of the solution was transferred to a glass HPLC vial containing a 250-glass insert. Fecal sample extracts for semi-polar metabolite (C18) analysis were reconstituted in 200 µL of a 90:10 H_2_O/acetonitrile solution and then vortexed until all material dissolved. The extracts were centrifuged at 12,000× g for 12 min at 4 °C, and 100 µL of the solution was transferred to a glass HPLC vial containing a 250 µL insert.

## General Mass Spectrometry Analytical Parameters

Polar and semi-polar metabolites were extracted using predominantly aqueous-based solvents and resolved using hydrophilic interaction liquid chromatography (HILIC) and reversed-phase columns, respectively. In contrast, lipids were extracted with an organic solvent and then resolved on a modified reversed-phase column. All metabolomic analyses were conducted on Thermo Fisher LC-MS/MS systems fitted with an Accela 1250 UHPLC pump system (Thermo Fisher Scientific, Waltham, MA, USA) coupled to a PAL autosampler (CTC Analytics AG., Zwingen, Switzerland) and either a QExactive or Exactive MS with electrospray ionisation. All three fecal analyses (polar, semi-polar, and lipid) and plasma lipid analyses were conducted on a Q-Exactive to facilitate MS/MS spectral identification. Plasma polar and semi-polar analyses were conducted on an Exactive due to instrument availability; thus, all subsequent data analyses described here were kept separate in their individual sample type (plasma or fecal) and analysis mode (polar, semi-polar, or lipid) rather than fully integrated. To ensure mass accuracy of each instrument and analysis mode, positive and negative mass calibrations using Pierce™ LTQ electrospray ionisation (ESI) (Positive and Negative Ion Calibration Solutions, Thermo Fisher Scientific, Waltham, MA, USA) of the Orbitrap systems were completed prior to sample analysis and after every 100 samples by direct infusion. The samples were cooled at 4 °C in an autosampler until sample injection. The samples were divided into three analytical batches, each comprising approximately 70 samples, with pooled QC and blank extracts injected every 10 samples. Multimodal analyses were conducted as previously described.^9, 10^ The LC-MS details are provided below. For annotation purposes, for the plasma lipids and all faecal extracts (all analysed on a QExactive), data-dependent MS/MS was performed on the pooled QC, and 10 randomly selected samples at the end of each batch of samples.

## Polar metabolomic liquid-chromatography mass spectrometry analysis

5 µL of plasma or fecal extract was injected into a 2 µL injection loop and eluted on SeQuant® ZIC®pHILIC column (100 x 2.1mm x 5µm, PEEK coated, Merck KGaA, Darmstadt, Germany), attached to a SeQuant® ZIC®-pHILIC Guard (20 x 2.1mm, PEEK coated, Merck KGaA, Darmstadt, Germany), and a KrudKatcher™ ULTRA HPLC In-Line Filter (0.004in x 0.5µm, Torrance, CA, USA). A 250 µL/min flow rate was maintained with solvent A 0.1% formic acid in acetonitrile and solvent B 16 mM ammonium formate in water. Gradient elution started at 3% B, increasing to 30% B at 12 min, 90% B at 14.50 min, and was held there until 18.50 min, at which point it returned to 3% B until 24 min for equilibration before the next sample injection. Mass spectral detection at 35,000 resolution for fecal extracts and 25,000 for plasma extracts was performed in positive and negative ionisation modes with ESI over 19 min, with a mass range from 55 to 825 *m/z*. For the fecal samples (analysed on a QExactive), data-dependant MS/MS was performed on the QC, and 10 randomly selected samples at the end of the batch to assist with annotation.

## Semi-polar metabolomic liquid-chromatography mass-spectrometry analysis

Five µL of plasma or fecal extract were injected into a 2 µL injection loop and eluted on a Hypersil

GOLD column (2.1 mm x 100 mm x 1.9 µm, Thermo Fisher Scientific, Waltham, MA, USA) with a 400 µL/min flow rate. Solvent A was 0.1% formic acid in water, and solvent B was 0.1% formic acid in acetonitrile. Gradient elution started at 0% B, increasing to 100% B at 11 min, held for 3 min and then back to 0% B at 14 min and held for 2 min for equilibration before the next sample injection. Mass spectral detection at 35,000 resolution for fecal and 25,000 resolution for plasma was performed in positive and negative ionisation modes with ESI over 14 min, with a mass range from 80 to 1200 *m/z*.

## Non-polar liquid-chromatography mass-spectrometry analysis

Five µL of plasma or fecal extract were injected into a 2 µL injection loop maintained at 65 °C with a flow rate of 600 µL/min and separated on an Acquity CSH™ C18 column 1.7 µm, 2.1 mm × 100 mm (Waters, Milford, MA, USA). Solvent A was 60% acetonitrile in water with 10 mM ammonium formate and 0.1% formic acid. Solvent B was 90% iso-propanol in acetonitrile with 10 mM ammonium formate and 0.1% formic acid. Gradient elution started at 15% B, increasing to 30% B at 2 min, 48% B at 2.50 min, 82% B at 11 min, then 99% B at 11.50, maintained until 14.10 min, reduced to 15% B and held there for 3 min for equilibration before the next sample injection. Mass spectral detection at 35,000 resolution was performed in positive and negative ionisation modes with ESI over 15 min and a mass range from 200 to 2000 *m/z*. Data-dependent MS/MS was performed on the QC and 10 randomly selected samples at the end of the batch to assist with lipid annotation.

# Targeted metabolomics

## Plasma amino acid

A tungstate precipitation protocol to analyze free proteogenic and non-proteogenic amino acids in EDTA plasma samples was carried out as described in Milan et al. (2015).^11^ Specifically, 20 µL of plasma was spiked with 15 µM L-Nor-Valine as internal standard, extracted with 20 µL 10% sodium tungstate and 160 µL of 0.04 M sulphuric acid. The mixture was incubated on ice for 3 min, then centrifuged at 12000× g for 10 minutes at 4 °C. 70 µL of 0.2 M borate buffer (pH 8.8) was added to the supernatant before adding 10 µL of AccQ-tag reagent (2.8 mg/ml in acetonitrile). In a sealed vial, the mixture was heated at 55 °C for 10 minutes before being subjected to ultra-performance liquid chromatography (UPLC). The UPLC system used a Thermo Scientific Dionex Ultimate 3000 pump, autosampler (maintained at 10 °C), column oven and fluorescence detector (set at Ex 250 nm, Em 395 nm) (Thermo Scientific, Dornierstrasse, Germany), and a Kinetex 1.7 µm C18 100A 100 x 2.1 mm column, preceded by a Krudkatcher inline filter (Phenomenex, Auckland, New Zealand) at 45°C. Mobile phase buffer, (80 mM sodium acetate, 3 mM triethylamine, 2.67 µM disodium calcium ethylenediaminetetraacetic acid) at pH 6.43 (obtained by addition of orthophosphoric acid), run with a complex gradient of acetonitrile from 2 to 17% (balance, water) over 24 minutes. Data were captured using Chromeleon 7.1 software (Thermo Scientific, Auckland, New Zealand). Standard curves were formulated for each compound within the physiological range of human plasma. Data reported in James et al., 2023.^12^

## Fecal bile acid

Extraction methods followed those previously described by Joyce et al. (2014),^13^ with minor modifications. Briefly, 100 mg of freeze-dried fecal samples were spiked with 100 ng of d4-CA and extracted with 700 µL ice-cold 50% MeOH in Eppendorf tubes pre-filled with 4 mm ceramic beads. The mixture was homogenized for six 30 s intervals (QIAGEN TissueLyser II, QIAGEN, Hilden,

Germany) and incubated at −20 ^o^C for 30 min and then centrifuged at 10,000× g for 25 min. Furthermore, 450 µL of the extract was transferred to a fresh tube and dried under nitrogen at 45 ^o^C. 1 mL of ice-cold ACN containing 5% formic acid was added to each tube, and the sample was briefly vortexed and agitated for 1 hour gently at room temperature. The mixture was centrifuged at 10,000× g for 10 min, and the resulting supernatant was transferred to Eppendorf tubes and dried under nitrogen at 45 ^o^C. The residual extract was dissolved in 150 µL of 50% MeOH, centrifuged at 10,000× g for 5

min, and transferred to glass vials for chromatographic analysis. The analysis was completed on a SCIEX LC-MS/MS QTRAP 6500+ system coupled to an ExionLC (SCIEX, Victoria, Australia).

Furthermore, 1 µL of the sample was injected into a Waters Aquity Ultra Performance Liquid Chromatography (UPLC) column (Massachusetts, USA) maintained at 50 °C with a flow rate of 300 µL/min. The mobile phase, solvent A, consisted of 10 mM ammonium formate in H_2_O, and solvent B, 10 mM ammonium formate, 5% ACN/95% MeOH. Gradient elution was as follows: 50% B held for 2 min, then increased to 87% B at 13.5 min, 99% B at 18 min, returning to 50% B at 19 min and held until 21 min for re-equilibration. Mass spectral detection was performed in negative electrospray ionization mode using multiple reaction monitoring (MRM) for 23 bile acid compounds and the internal standard using electrospray ionization. Standards for all target compounds were run prior to sample analysis to optimize MRM conditions and separation of compounds. The source voltage was set to 4500 V, with a source temperature of 550 °C. Data was captured using Analyst (V1.6) software and processed on MultiQuant (V3.0.2) SCIEX software. Bile acid concentrations were generated from standard curves of standard injections for all 23 bile acids and the deuterated internal standard (d4-CA). Concentrations of bile acids were corrected to the dry weight of fecal matter and are presented as µg/mg of dried fecal sample. Data reported in James et al. (2021).^14^

## Fecal short-chain fatty acids

Fecal samples were stored at −80 °C, and short-chain fatty acids (SCFA, 14 linear and branched (C1 SCFA through to C7 SCFA)) were measured using an MS-probe and stable isotope coding LCMS method with modifications.^15, 16^ This method uses ^12^C/^13^C_6_-3-nitrophenylhydrazine (3NPH) to quantitatively convert SCFAs to their 3-nitrophenylhydrazones to increase their analysis sensitivity. Isotope label coding was enabled using ^13^C_6_-3NPH to create an internal standard (IS) for each SCFA.

In brief, to each sample (10 µL from a solution of 250 mg fresh weight fecal material dissolved in 1.5 mL 50:50 CH_3_CN:H_2_O) in an individual well of a 2 mL 96-deepwell plate (Phenomenex, Torrance, CA, USA) was added 3-NPH (20 µL; 200 mM in 75:25 methanol/water v/v), EDC-6% pyridine (20 µL; 120 mM in methanol) and 75:25 methanol/water v/v (10 µL). Standards (20 µL) were treated similarly, but without the addition of the 75:25 methanol/water v/v (10 µL). For higher level standards (2-40 mM), the volume of the derivatising solutions was increased to 40 µL. Matrix spikes were prepared at physiologically relevant concentrations on each plate. The plate was sealed with a silicone mat

(Phenomenex, Torrance, CA, USA) and reacted at RT with agitation using a ThermoMixer^®^ C (Eppendorf, Hamburg, Germany) at 1000 rpm for 45 min. The reaction was quenched by the addition of quinic acid (20 µL; 200 mM in 75:25 methanol/water v/v), and samples, standards and matrix spikes diluted with 10% aqueous methanol to give a total volume of 1 mL. A 100 µL aliquot of each sample, standard and matrix spike was mixed with 100 µL of the IS mix in a new 96-well plate, and a 1 µL aliquot was injected for LC-MS/MS.

LC-MS experiments were carried out on a 5500 QTrap triple quadrupole/linear ion trap (QqLIT) mass spectrometer equipped with a Turbo V™ ion source and atmospheric pressure chemical ionisation (APCI) probe (AB Sciex, Concord, ON, Canada) coupled to an Ultimate 3000 UHPLC (Dionex, Sunnyvale, CA, USA). Chromatographic separation was performed on an Acquity UPLC® CSH™ C18

(2.1 x 150 mm, 1.7 µm) column (Waters, Dublin, Ireland), using water (solvent A) and acetonitrile (solvent B) as the mobile phase for gradient elution. The column flow rate was 0.4 mL min^-1^; the column temperature was 65 °C, and the autosampler was kept at 5 °C. The initial mobile phase, 0.5% B, was ramped linearly to 2.5% B at 3 min, then 17% B at 6 min, 45% B at 10 min, 55% B at 13 min, 100% B between 14 and 18 min before resetting to the original conditions.

MS data were acquired in the negative mode using a multiple reaction monitoring method with Analyst

1.6 software and processed with MultiQuant 3.0.2 software (AB Sciex, Concord, ON, Canada).

**References**

1. Zhang J, Kobert K, Flouri T, Stamatakis A. PEAR: a fast and accurate Illumina Paired-End reAd mergeR. Bioinformatics 2013; 30:614-20. doi: 10.1093/bioinformatics/btt593.
2. Bengtsson-Palme J, Hartmann M, Eriksson KM, Pal C, Thorell K, Larsson DG, et al. METAXA2: improved identification and taxonomic classification of small and large subunit rRNA in metagenomic data. Mol Ecol Resour 2015; 15:1403-14. doi: 10.1111/1755-0998.12399.
3. Quast C, Pruesse E, Yilmaz P, Gerken J, Schweer T, Yarza P, et al. The SILVA ribosomal RNA gene database project: Improved data processing and web-based tools. Nucleic Acids Res 2012; 41:D590-D6. doi: 10.1093/nar/gks1219.
4. Buchfink B, Xie C, Huson DH. Fast and sensitive protein alignment using DIAMOND. Nat Methods 2015; 12:59-60. doi: 10.1038/nmeth.3176.
5. Huson DH, Beier S, Flade I, Górska A, El-Hadidi M, Mitra S, et al. MEGAN community edition-interactive exploration and analysis of large-scale microbiome sequencing data. PloS Comput Biol 2016; 12:e1004957. doi: 10.1371/journal.pcbi.1004957.
6. Fraser K, James SC, Young W, Gearry RB, Heenan PE, Keenan JI, et al. Characterisation of the plasma and faecal metabolomes in participants with functional gastrointestinal disorders. Int J Mol Sci 2024; 25:13465. doi: 10.3390/ijms252413465.
7. Xu J, Begley P, Church SJ, Patassini S, Hollywood KA, Jüllig M, et al. Graded perturbations of metabolism in multiple regions of human brain in Alzheimer's disease: Snapshot of a pervasive metabolic disorder. Biochim Biophys Acta-Mol Basis Dis 2016; 1862:1084-92. doi:

10.1016/j.bbadis.2016.03.001.

1. Hosseinkhani F, Dubbelman A-C, Karu N, Harms AC, Hankemeier T. Towards Standards for Human Fecal Sample Preparation in Targeted and Untargeted LC-HRMS Studies. Metabolites 2021; 11:364. doi: 10.3390/metabo11060364.
2. Bassett SA, Young W, Fraser K, Dalziel JE, Webster J, Ryan L, et al. Metabolome and microbiome profiling of a stress-sensitive rat model of gut-brain axis dysfunction. Sci Rep 2019; 9:14026. doi: 10.1038/s41598-019-50593-3.
3. Fraser K, Lane GA, Otter DE, Hemar Y, Quek S-Y, Harrison SJ, et al. Analysis of metabolic markers of tea origin by UHPLC and high resolution mass spectrometry. Food Res Int 2013; 53:82735. doi: 10.1016/j.foodres.2012.10.015.
4. Milan A, D’Souza R, Pundir S, Pileggi C, Barnett M, Markworth J, et al. Older adults have delayed amino acid absorption after a high protein mixed breakfast meal. J Nutr Health Aging 2015; 19:839-45. doi: 10.1007/s12603-015-0500-5.
5. James SC, Fraser K, Cooney J, Günther CS, Young W, Gearry RB, et al. Concentrations of plasma amino acids and neurotransmitters in participants with functional gut disorders and healthy controls. Metabolites 2023; 13:313. doi: 10.3390/metabo13020313.
6. Joyce SA, MacSharry J, Casey PG, Kinsella M, Murphy EF, Shanahan F, et al. Regulation of host weight gain and lipid metabolism by bacterial bile acid modification in the gut. Proc Natl Acad Sci U S A 2014; 111:7421-6. doi: 10.1073/pnas.1323599111.
7. James SC, Fraser K, Young W, Heenan PE, Gearry RB, Keenan JI, et al. Concentrations of fecal bile acids in participants with functional gut disorders and healthy controls. Metabolites 2021; 11:612. doi: 10.3390/metabo11090612.
8. Han J, Lin K, Sequeira C, Borchers CH. An isotope-labeled chemical derivatization method for the quantitation of short-chain fatty acids in human feces by liquid chromatography–tandem mass spectrometry. Anal Chim Acta 2015; 854:86-94. doi: 10.1016/j.aca.2014.11.015.
9. Fristedt R, Ruppert V, Trower T, Cooney J, Landberg R. Quantitation of circulating short-chain fatty acids in small volume blood samples from animals and humans. Talanta 2024; 272:125743. doi:

10.1016/j.talanta.2024.125743.
